# Informed Dictionary‐Guided Monte Carlo Inversion for Robust and Reproducible Multidimensional MRI

**DOI:** 10.1002/mrm.70228

**Published:** 2025-12-28

**Authors:** Joon Sik Park, Eppu Manninen, Yihong Yang, Dan Benjamini

**Affiliations:** ^1^ Multiscale Imaging and Integrative Biophysics Unit National Institute on Aging, National Institutes of Health Baltimore Maryland USA; ^2^ Neuroimaging Research Branch National Institute on Drug Abuse, National Institutes of Health Baltimore Maryland USA

**Keywords:** brain microstructure, dictionary matching, diffusion and relaxation, MC inversion, multidimensional MRI

## Abstract

**Purpose:**

To develop a robust and efficient multidimensional MRI (MD‐MRI) data processing framework for accurately estimating joint frequency‐dependent diffusion‐relaxation distributions, while overcoming computational limitations and noise instability inherent to Monte Carlo (MC) inversion.

**Methods:**

We introduced an Informed Dictionary‐guided Monte Carlo (ID‐MC) strategy that incorporates data‐driven dictionary matching into the inversion process, followed by targeted local mutation refinement to enhance flexibility and reduce overfitting. This hybrid approach aims to improve the stability, accuracy, and reproducibility of MD‐MRI parameter estimation. We evaluated ID‐MC through in silico simulations across a range of signal‐to‐noise ratios and in vivo test–retest experiments in the human brain. Reproducibility was assessed using intraclass correlation coefficients (ICC) and within‐subject variability, allowing rigorous comparison with MC.

**Results:**

In simulations, the ID‐MC approach consistently achieved lower fitting errors and higher estimation accuracy across a wide range of noise levels, demonstrating its ability to balance local flexibility and global biological plausibility. Compared to MC inversion, ID‐MC also reduced computation time by approximately 69%, highlighting its potential for time‐efficient large‐scale applications. In in vivo test–retest analyses, ID‐MC substantially improved reproducibility, doubling the number of MD‐MRI parameters with ICC greater than 0.75 relative to MC. Notably, diffusion frequency‐dependent parameters, previously poorly reproducible with MC, showed up to 146% higher ICC with ID‐MC.

**Conclusion:**

By integrating data‐driven dictionary matching with targeted mutation refinement, ID‐MC improves the robustness, reproducibility, and computational efficiency of MD‐MRI inversion, supporting studies that require highly sensitive detection of subtle brain microstructural changes.

## 1 Introduction

Water diffusion in biological tissues is shaped by interactions with complex cellular structures, including membranes, organelles, and macromolecular environments. Diffusion magnetic resonance imaging (dMRI) captures these dynamics by measuring water displacement at the micrometer scale, providing an indirect yet powerful probe of tissue microarchitecture [1]. In brain tissue, the dMRI signal from a voxel reflects the combined contributions of multiple water populations—axons, dendrites, somas, extracellular space, and glia—each with distinct diffusion properties determined by morphology and permeability. Pathological changes such as demyelination, edema, or swelling further increase signal complexity and hinder interpretation.

To enhance microstructural specificity, dMRI can be complemented by relaxation parameters such as longitudinal ($R_{1}$) and transverse ($R_{2}$) relaxation rates, which reflect the local biochemical environment, magnetization exchange, and macromolecular content [2, 3]. However, when analyzed independently, diffusion and relaxation provide only a partial view of tissue properties and may overlook the inherently multidimensional nature of intra‐voxel heterogeneity.

Traditional voxel‐wise models often assume spatial homogeneity, which limits their ability to resolve the intra‐voxel mixtures of cell types, shapes, and orientations. To overcome this, multidimensional MRI (MD‐MRI) techniques have been developed to jointly encode diffusion and relaxation properties in a unified framework [4]. By increasing data dimensionality, MD‐MRI captures interdependencies between structural and compositional features, thereby improving microstructural specificity [5, 6, 7, 8, 9, 10, 11] and sensitivity to pathology [12, 13, 14, 15, 16].

Historically, models like diffusion tensor imaging (DTI) [17] provided the first quantitative link between dMRI signals and tissue structure. However, these models rely on simplifying assumptions that may not hold in heterogeneous or pathological tissue [18]. Biophysical models with multiple compartments have since been introduced to improve specificity, particularly in white matter (WM) [10, 19, 20, 21, 22]. While these models enhance interpretability, they often rely on fixed assumptions or parameters that reduce flexibility and may introduce errors in complex conditions [23, 24, 25].

As an alternative, data‐driven, biophysical model‐free methods directly estimate the distribution of diffusion and relaxation parameters from the signal itself. Originating from early $T_{2}$ distribution studies in muscle [26], these nonparametric approaches have advanced to extract joint diffusion‐relaxation spectra that reflect distinct water environments and their correlations [15, 27, 28, 29, 30, 31, 32, 33]. Importantly, these methods do not require prior assumptions about compartment number or geometry. When combined with advanced diffusion encoding strategies–such as tensor‐valued gradients with modulated frequency content [34]–they allow precise control over the frequency content of the diffusion sensitization and offer access to frequency‐dependent diffusion spectra, D(ω) [35, 36].

Frequency‐domain representations of diffusion, which relate to velocity autocorrelation, are valuable for studying biological systems with restricted and time‐dependent water motion [37, 38]. The frequency‐dependent diffusion tensor distribution enables separation of water pools and their diffusion time dependencies, allowing microstructural metrics to be estimated without strong priors [39]. This flexibility is essential for resolving fine tissue heterogeneity and detecting subtle pathology.

A recent study has demonstrated that high‐dimensional $D(\omega )-R_{1}-R_{2}$ distributions can be mapped across the human brain in vivo using sparsely sampled MD‐MRI protocols [40]. With optimized gradient design and robust reconstruction, intra‐voxel distribution moments—means, variances, and covariances—can be recovered to distinguish WM, gray matter (GM), and cerebrospinal fluid (CSF) based on their unique multidimensional signatures [41].

Despite these advances, estimating accurate parameter distributions from MD‐MRI remains an ill‐posed problem [42]. The Monte Carlo (MC) inversion [43] has been widely used to address this challenge but is often computationally intensive and sensitive to noise [44], limiting the scalability of MD‐MRI in large‐scale studies.

A prior test–retest study evaluated the reliability of MD‐MRI metrics from $D(\omega )-R_{1}-R_{2}$ distributions [45]. While ROI‐level metrics—such as mean diffusivity, anisotropy, and $R_{2}$—were repeatable, higher order and frequency‐dependent measures showed lower reproducibility, likely due to noise sensitivity and limited sampling. These findings suggested that, despite the rich microstructural information, the robustness of some parameters remains sub‐optimal, particularly when targeting low‐signal components.

To address these limitations, we propose a new MD‐MRI processing framework that extends the MC inversion method. This framework integrates a data‐driven informed dictionary matching step with targeted local mutation refinement to improve the stability of parameter estimation. We evaluate the proposed approach both in silico, using simulations designed to reflect biologically realistic conditions across varying signal‐to‐noise ratios (SNRs), and in vivo, by assessing the test–retest reproducibility of MD‐MRI parameters in the human brain.

## 2 Theory

### 2.1 Signal Model in Multidimensional MRI

In diffusion‐relaxation MD‐MRI, voxel‐level microstructure is represented by the joint distribution of frequency‐dependent diffusion tensors D(ω) and relaxation parameters $R_{1}$ and $R_{2}$, denoted as $P(D(\omega ),R_{1},R_{2})$. For numerical implementation, this continuous distribution is approximated by a discrete vector w, where each element is the weight of a distinct diffusion‐relaxation component. The observed signal s is modeled as a weighted sum of these components under a given acquisition setting, as follows:

(1) $$\begin{array}{cc} s_{m}[b_{m}(\omega ),{\text{TE}}_{m},{\text{TR}}_{m}] & =\overset{N_{c}}{\underset{n=1}{\sum }}w_{n}\\ & \;\exp\left(-\int_{-\infty }^{\infty }b_{m}(\omega ):D_{n}(\omega )\;d\omega \right)\\ & \;\times \left[1-\exp\left(-{\text{TR}}_{m}\cdot {R_{1}}_{,n}\right)\right]\\ & \;\times \exp\left(-{\text{TE}}_{m}\cdot {R_{2}}_{,n}\right), \end{array}$$

where the colon denotes a generalized scalar product, m is the acquisition index, $w_{n}$ the weight of the nth component, and $N_{c}$ the total number of intravoxel components. Each component contributes to the signal according to $w_{n}$, representing the fraction of signal from the nth diffusion‐relaxation component. The kernel function k captures the combined effects of the diffusion spectra D(ω) and relaxation rates $R_{1}$ and $R_{2}$, and is given by:

(2) $$\begin{array}{cc} k_{m}[{R_{1}}_{n},{R_{2}}_{n},D_{n}(\omega )] & =\exp\left(-\int_{-\infty }^{\infty }b_{m}(\omega ):D_{n}(\omega )\;d\omega \right)\\ & \;\times \left[1-\exp\left(-{\text{TR}}_{m}\cdot {R_{1}}_{,n}\right)\right]\\ & \;\times \exp\left(-{\text{TE}}_{m}\cdot {R_{2}}_{,n}\right), \end{array}$$

where the acquisition‐specific encoding parameter $b_{m}(\omega )$ is known and therefore omitted from $k_{m}$'s arguments for notational simplicity. The kernel function is defined by the imaging sequence and the target parameters. In this study, a 2D single‐shot spin‐echo EPI sequence is employed, and the kernel is derived from the corresponding spin‐echo MR signal equation. The inversion of Equation (1) is facilitated by approximating $D_{n}(\omega )$ as an axi‐symmetric Lorentzian spectrum [39, 46]. The diffusion and relaxation parameters to be estimated, $\left(D_{n}(\omega ),R_{1,n},R_{2,n}\right)$, are represented as:

(3) $$\begin{array}{cc} (D_{n}(\omega ),{R_{1}}_{,n},{R_{2}}_{,n})=( & {D_{\|}}_{n},{D_{⊥}}_{n},\theta_{n},\phi_{n},{D_{0}}_{n},\\ & {\Gamma_{\|}}_{n},{\Gamma_{⊥}}_{n},{R_{1}}_{n},{R_{2}}_{n}), \end{array}$$

where $D_{\|}$ and $D_{⊥}$ are the axial and radial diffusivity at zero frequency, θ and ϕ define the orientation of the diffusion tensor, $D_{0}$ denotes the high‐frequency isotropic diffusivity, and $\Gamma_{\|}$ and $\Gamma_{⊥}$ are the axial and radial spectral transition frequencies.

Given all the above considerations, the signal equation in Equation (1) can be written in matrix form as:

(4) s=K·w,

where $s={\left[s_{1},s_{2},\ldots ,s_{M}\right]}^{⊤}$ is the signal vector across *M* acquisitions, $K\in {\mathbb{R}}^{M\times N_{c}}$ is the kernel matrix whose columns represent the signal responses of $N_{c}$ diffusion‐relaxation components, and $w={\left[w_{1},w_{2},\ldots ,w_{N_{c}}\right]}^{⊤}$ is the vector of non‐negative weights representing the contribution of each component.

### 2.2 Monte Carlo Inversion

With incomplete MD‐MRI measurements, the inverse problem in Equation (4) becomes ill‐conditioned, hindering accurate parameter estimation [42, 47]. Several computational strategies have been proposed to address this, including $\ell_{1}$ [48] and $\ell_{2}$ [49] regularization, maximum entropy [50], and MC methods [51]. MC inversion has emerged as an effective approach for sparsely sampled MD‐MRI data [43], estimating parameter distributions through iterative sampling and refinement, with demonstrated success in complex systems [9, 40, 41, 52]. The standard MC framework comprises the following three steps:

1. *Bootstrap Sampling*: MC inversion begins with bootstrap sampling, a resampling technique that draws signal subsets with replacement to reflect measurement noise and variability. This broadens the solution space and supports robust estimation of parameter distributions, enhancing stability and reproducibility [44].
2. *Proliferation*: Each bootstrap sample undergoes proliferation to globally explore the parameter space by generating candidate sets (e.g., frequency‐dependent tensors and relaxation parameters; Equation 3). Kernel vectors are computed (Equation 2) and fitted via NNLS to estimate weights w; sets with non‐zero weights are retained while redundant ones are pruned.
3. *Mutation*: Mutation refines top parameter sets through small stochastic perturbations. The perturbed sets are evaluated via kernel vectors (Equation 2) and corresponding NNLS weights w, selectively retaining the best fits to improve accuracy and local exploration.

For each voxel, parameter sets from Equation (3) are aggregated to characterize underlying diffusion and relaxation distributions.

### 2.3 Informed Dictionary‐Guided MC (ID‐MC) Inversion

While MC inversion effectively models complex MD‐MRI signals, it is computationally demanding and susceptible to instability and overfitting, particularly at low SNR or in redundant parameter spaces [44]. Moreover, its reliance on random sampling during the search process can lead to inefficiencies and challenges in reproducibility. To address these limitations, we propose a reformulated inversion framework that replaces stochastic proliferation with a data‐driven, dictionary‐guided approach.

This approach assumes that similarity in the kernel subspace corresponds to proximity in parameter space. Rather than generating random candidates, it uses an informed dictionary of precomputed kernel–parameter pairs derived from training data spanning a wide age range. For each bootstrap signal, the closest dictionary entry is selected via cosine similarity, followed by NNLS weighting and local refinement through mutation. A limitation arises when patient data contain abnormalities not represented in the dictionary. However, under the core assumption that kernel‐space similarity reflects parameter‐space proximity, the method should remain broadly robust, offering a stable and biologically plausible subspace for inversion even with moderate deviations. Given these considerations, we hypothesize that the hybrid strategy retains the robustness of bootstrap‐based inversion while improving efficiency, stability, and reproducibility. Thus, the proposed method consists of the following key steps:

1. *Informed Dictionary Generation*: Leverage a representative training set to build an informed dictionary.
2. *Signal Pattern Matching*: For each bootstrap signal, the most similar dictionary entry is identified using cosine similarity.
3. *Projection*: The matched kernel vectors are used in NNLS fitting to estimate the corresponding parameter set.
4. *Mutation*: Apply local perturbations to refine the estimates, improving fitting accuracy.

**FIGURE 1:**
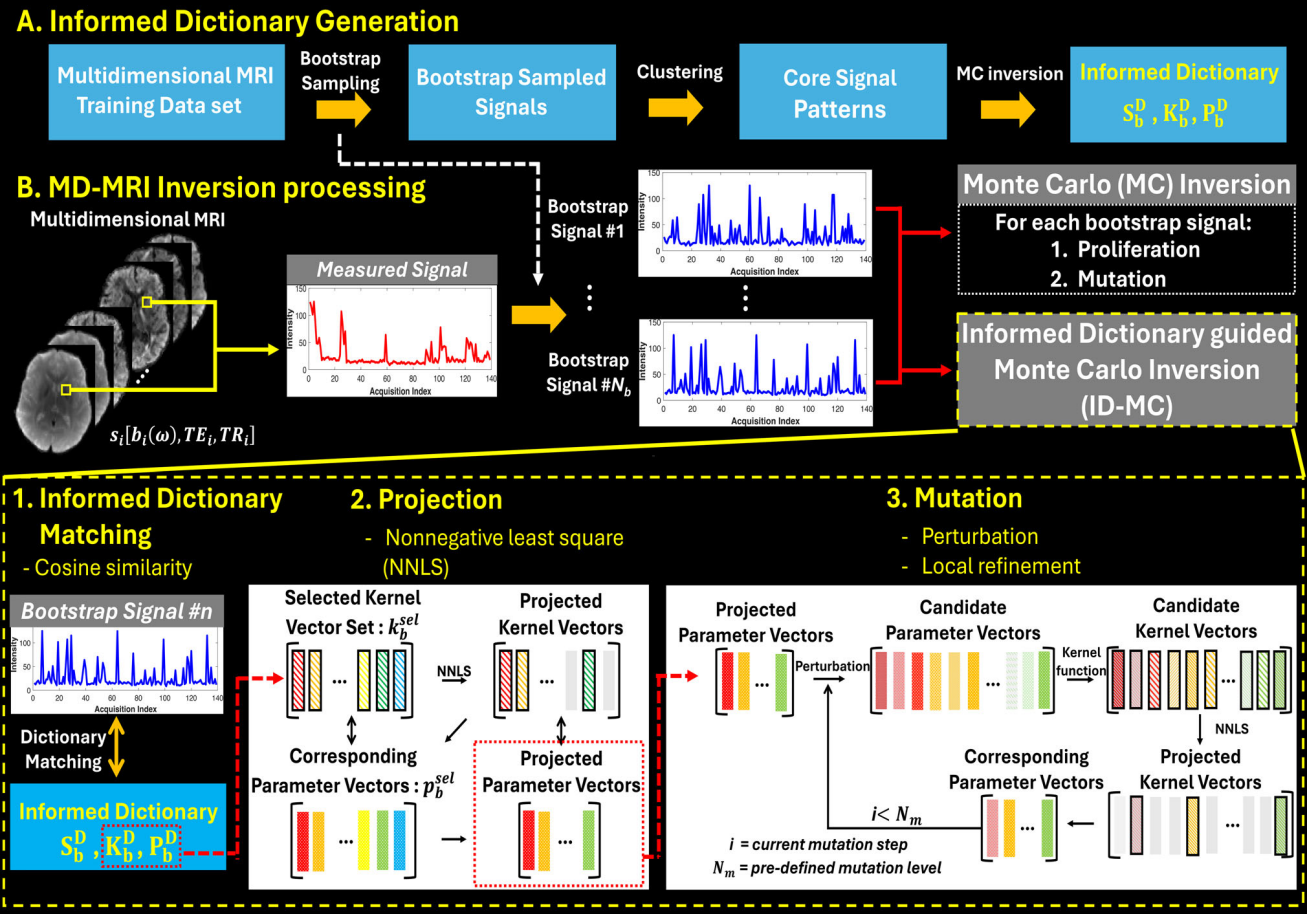
Overview of the Informed Dictionary‐guided Monte Carlo (ID‐MC) inversion framework for MD‐MRI. (A) Bootstrap‐sampled signals from training data are clustered to construct the informed dictionary, consisting of signal patterns ($S_{b}^{D}$), kernel sets ($K_{b}^{D}$), and corresponding parameter sets ($P_{b}^{D}$). (B) For each test bootstrap signal $s_{i}$, MC inversion consisting of proliferation and mutation steps is performed. Alternatively, the ID‐MC inversion performs matching with the informed dictionary, projects the signal onto the selected kernel space using nonnegative least squares (NNLS), and applies local refinement through parameter mutation. This process iteratively updates the kernel set and weights to estimate the accurate parameter set.

A schematic of the ID‐MC inversion framework is shown in Figure 1, with theoretical and practical details provided in the following subsections.

#### 2.3.1 Informed Dictionary Generation

The informed dictionary comprises three components:

1. a bootstrap signal dictionary $S_{b}^{D}$;
2. a kernel vector dictionary representing the constituent kernels of each signal $K_{b}^{D}$; and
3. the parameter sets used to generate the kernel vectors $P_{b}^{D}$, where subscript b denotes the bootstrap index.

Compared with magnetic resonance fingerprinting [53] or biophysical modeling, constructing a signal dictionary and parameter table in MD‐MRI is particularly challenging. As shown in Equation (3), the parameter space is high‐dimensional, adding substantial complexity. Signals are formed by combining kernel vectors (Equation 2) derived from complex parameter sets, and the number of kernel vectors per signal is variable with only an upper bound. This leads to highly diverse, high‐dimensional signal representations. In addition, the weighting vector w reflects the underlying parameter distribution, making it difficult to synthesize realistic signals with controlled parameters. These factors present major obstacles for conventional dictionary construction.

In this work, we constructed a data‐driven informed dictionary using a training set of 10 normative subjects with balanced sex and varied ages (see Section 3.2). Brain voxels from all training subjects were pooled and clustered by signal similarity to reduce redundancy while preserving representative patterns. To introduce statistical variation while maintaining distributional characteristics, bootstrap sampling was applied to the clustered signals. Each resampled signal underwent MC inversion to generate $K_{b}^{D}$ and $P_{b}^{D}$, from which $S_{b}^{D}$ was synthesized using Equation (4).

#### 2.3.2 Signal Pattern Matching

To utilize the informed dictionary in the proposed inversion, we use the same bootstrap sampling indices from dictionary construction to generate signal realizations from the input data. Each bootstrap signal is matched to entries in $S_{b}^{D}$ via pattern matching.

This step identifies the signal vector in $S_{b}^{D}$ that best aligns with the input using cosine similarity, which is robust to magnitude differences by emphasizing directional similarity—crucial in MD‐MRI, where signal shape and orientation often carry more information than absolute intensity. The cosine similarity between the ith bootstrap signal and the jth dictionary vector is computed as:

(5) $$C_{\text{sim}}(i,j)=\frac{s_{i}^{b}\cdot s_{j}^{d}}{\left\|s_{i}^{b}\|\|s_{j}^{d}\right\|},$$

where $s_{i}^{b}$ is the bootstrap signal from the i‐th voxel, and $s_{j}^{d}$ is the j‐th dictionary vector. The most similar dictionary signal is selected as:

(6) $$s_{i}^{\text{sel}}=s_{j^{*}}^{d},\;j^{*}=\arg\underset{j}{\max}\;C_{\text{sim}}(i,j)$$

The selected signal $s_{i}^{\text{sel}}$ is used to retrieve the corresponding kernel and parameter sets, $k_{b}^{\text{sel}}\in K_{b}^{D}$ and $p_{b}^{\text{sel}}\in P_{b}^{D}$, which are then used in the projection step to estimate weights.

#### 2.3.3 Projection

We assume the input bootstrap signal can be expressed as a linear combination of kernel vectors $k_{b}^{\text{sel}}$ from the matched dictionary entry. The corresponding weights are estimated using kernel‐constrained NNLS, ensuring non‐negative and physically interpretable diffusion‐relaxation weights. The relationship is given by:

(7) $$s_{i}^{b}\approx k_{\text{b}}^{\text{sel}}\cdot w^{\prime },$$

where $k_{\text{b}}^{\text{sel}}$ is the selected kernel set and $w^{\prime }$ is the estimated weight vector obtained by solving:

(8) $$\arg\underset{w^{\prime }\geq 0}{\min}\;\|s_{i}^{b}-k_{\text{b}}^{\text{sel}}\cdot w^{\prime }{\|}^{2}.$$

This yields an ensemble of bootstrap solutions with estimated weights and matched parameters from $k_{b}^{\text{sel}}$. We define this approach, which solves solely based on the informed dictionary, as the direct Dictionary Matching (DM) inversion.

#### 2.3.4 Mutation

While DM inversion offers an efficient initial estimate of the parameter distribution, its reliance on a single matched kernel set from training data limits flexibility and may lead to underfitting. When accumulated across bootstrap realizations, this can introduce systematic bias. To address this, we incorporate a mutation step to locally refine the selected kernel candidates. Each parameter vector $p_{j}$ from the matched set is perturbed by small stochastic variations sampled from a zero‐mean Gaussian distribution, where the perturbation scale σ defines the exploration radius:

(9) $${\tilde{p}}_{j}^{\left(r\right)}=p_{j}+\epsilon_{j}^{\left(r\right)},\;\epsilon_{j}^{\left(r\right)}∼𝒩(0,\sigma^{2}I),\;r=1,\ldots ,R$$

Each perturbed set ${\tilde{p}}_{j}^{\left(r\right)}$ is then used to generate a kernel vector via Equation (2). These, combined with the original matched kernels, form an expanded kernel set:

(10) $$K_{i}^{\text{mut}}=\left[K_{b}^{\text{sel}},{\tilde{K}}_{b}^{\left(1\right)},\ldots ,{\tilde{K}}_{b}^{\left(R\right)}\right].$$

A second NNLS fit is then performed to refine weights:

(11) $$w^{*}=\arg\underset{w\geq 0}{\min}\;{\left\|s_{i}^{b}-K_{i}^{\text{mut}}\cdot w\right\|}^{2}.$$

By locally exploring the parameter space around the matched candidates, mutation refines signal fitting, reduces underfitting, and improves robustness. Unlike MC inversion, which applies mutation after broad proliferation, our method initiates mutation directly from the DM‐derived kernel set, enabling more targeted local exploration. We refer to this hybrid strategy as Informed Dictionary‐Guided MC (ID‐MC) inversion.

## 3 Methods

### 3.1 Participants

Healthy adult participants were recruited from ongoing volunteer cohorts at the National Institute on Drug Abuse (NIDA). All procedures were approved by the local Institutional Review Board, and written informed consent was obtained. Prior to imaging, standard screening was performed, including physical exams and health history questionnaires. Individuals with major medical, neurological, psychiatric, or substance use histories were excluded. Two data sets were used:

1. *Training set*: Ten healthy adults (5 male, 5 female; ages 23–77) were selected to maximize age diversity.
2. *Test‐retest set*: Ten adults (average age 48 ± 14.4 years; 4 female) underwent two MRI sessions several weeks apart, yielding 20 scans.

### 3.2 Data

#### 3.2.1 Acquisition Scheme

To enable efficient and sparse MD‐MRI sampling, the acquisition scheme followed heuristics from prior studies [40, 41, 45], using numerically optimized gradient waveforms [54] to generate b‐tensors with distinct shapes—linear ($b_{\Delta }=1$), spherical ($b_{\Delta }=0$), and planar ($b_{\Delta }=-0.5$)—and amplitudes ranging from 0.1–3 ms/μm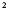. One non‐diffusion‐weighted volume (b=0 ms/μm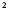) was included. Waveforms sampled centroid frequencies between 6.6 and 21 Hz ($\omega_{cent}/2\pi$). Sensitivity to $R_{1}$ and $R_{2}$ relaxation was achieved by varying TR (0.62–7.6 s) and TE (40–150 ms). The final protocol included 139 unique diffusion‐relaxation volumes. Full acquisition details are in Johnson et al. [40] and summarized in Figure S1 and Table S1.

#### 3.2.2 Numerical Simulation

To simulate the system, a set of ground‐truth microstructural and relaxation parameters—$\left[D_{\|},D_{⊥},\theta ,\phi ,D_{0},\Gamma_{\|},\Gamma_{⊥},R_{1},R_{2}\right]$ (Table 1)—was generated and used to compute signal descriptors and the ground‐truth dataset $S_{gt}$ via the decay model in Equation (1). These were selected to mimic a complex microstructural scenario of partial volume comprised of water in soma (Comp. I), crossing fibers (Comp. II and III), and CSF (Comp. IV). This approach follows established practices for multidimensional “Laplace” inversion [9], which remains ill‐posed even in the absence of noise, thus requiring regularization [4, 42, 49]. Noise was simulated by adding Rician noise to $S_{\text{gt}}$:

(12) $$S_{i}=\sqrt{{\left(S_{gt}+\frac{v_{i}}{\text{SNR}}\right)}^{2}+{\left(\frac{v_{i}^{\prime }}{\text{SNR}}\right)}^{2}},$$

where $v_{i}$ and $v_{i}^{\prime }$ are zero‐mean Gaussian samples.

**TABLE 1:** Ground truth simulation MD‐MRI components.

Parameter	$D_{\|}$	$D_{⊥}$	θ	ϕ	$D_{0}$	$\Gamma_{\|}$	$\Gamma_{⊥}$	$R_{1}$	$R_{2}$	
Comp.	[ms/μm 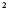 ]	[ms/μm 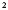 ]	[°]	[°]	[ms/μm 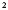 ]	[Rad/s]	[Rad/s]	[1/s]	[1/s]	w
I	0.5	0.5	0	0	2	250	250	1	13	0.35
II	2.5	0.1	78	78	2	200	700	1.5	20	0.25
III	2.5	0.1	78	23	2	200	700	1.5	20	0.25
IV	3	3	0	0	3	10 000	10 000	0.3	6	0.15

To simultaneously simulate realistic variability and evaluate model robustness to bias, we introduced controlled perturbations relative to the ground truth to generate a series of biased training dictionaries. The ID‐MC training data sets were constructed based on the component values listed in Table 1, with added Gaussian noise levels with $\sigma_{\text{bias}}=[0.2,0.3,0.4,0.5]$, to create multiple levels of biased training sets (Table S2). Parameter variations were introduced to model biological variability, simulate differences between training and testing data, and assess method robustness. For each bias level, a training dictionary was generated and augmented with Rician noise to produce 40,000 signals (10,000 per SNR: 130, 90, 60, 30). The test set was similarly noised to yield 10,000 signals per SNR. Each biased dictionary was then used to invert its corresponding noisy signals, and the reported results reflect these bias‐controlled experiments.

Inversion accuracy was evaluated using the Earth Mover's Distance (EMD) [55], a validated metric for comparing multidimensional distributions in MD‐MRI [7, 40].

#### 3.2.3 In Vivo Human

Human brain MD‐MRI was acquired on a 3T Prisma scanner (Prisma, Siemens Healthcare, Erlangen, Germany) with a 32‐channel head coil. A 2D single‐shot spin‐echo EPI sequence with tensor‐valued diffusion encoding was used, employing numerically optimized waveforms [41, 56]. Imaging protocol employs 2 mm isotropic voxels, a 228×228×110 mm^3^ FOV, 1512 Hz/Px bandwidth, GRAPPA (factor 2), 0.8 ms echo spacing, and 0.75 partial Fourier. The data sets were acquired with a single‐phase encoding direction (anterior to posterior, AP), and an additional b = 0 ms μm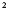 volume with reversed phase encoding direction (PA). A total of 139 measurements were acquired over 40 min. Additionally, a fat‐suppressed 1 mm isotropic T1‐weighted MPRAGE scan was acquired for registration and segmentation. The SNR (center b=0 divided by background standard deviation) was 136±14.

Preprocessing was conducted using TORTOISE [57, 58], including MPPCA denoising [59], Gibbs correction [60, 61], and motion/eddy correction with DIFFPREP [62]. Susceptibility distortion was corrected by converting T1W to T2W‐like b=0 contrast [63] and applying DRBUDDI [64]. Final images were interpolated once to anatomical space at native in‐plane resolution.

### 3.3 Inversion Implementation

We evaluated MC, DM, and ID‐MC inversions across varying mutation steps ($N_{m}$). All methods invert Equation (1) using parameters from Equation (3), sampled within biologically plausible bounds: $0.05\leq D_{||/⊥/0}\leq 5$ FDμm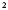/ms, 0≤Θ≤π, 0≤Φ≤2π, $0.2\leq R_{1}\leq 2s^{-1}$, $1\leq R_{2}\leq 30s^{-1}$, and $0.01\leq \Gamma_{||/⊥}\leq 10000\;s^{-1}$. MC inversion was performed with $N_{p}=20$ proliferation steps, $N_{m}=20$ mutation steps, up to $N_{c}=10$ components, and $N_{b}=100$ bootstrap rounds. Source code is available at https://github.com/jan‐martin‐mri/md‐dmri.

To generate the informed dictionary, each bootstrap set $S_{b}^{D}$ ($N_{b}=100$) contained 1.2 million signal patterns from all brain voxels in the training set. The corresponding kernels $K_{b}^{D}$ and parameter sets $P_{b}^{D}$ were generated using the same $N_{p}=20$ and $N_{m}=20$ settings as MC inversion.

ID‐MC was implemented in MATLAB R2023a (MathWorks) by modifying the Diffusion MRI Toolbox. Each parameter set was perturbed using random Gaussian‐distributed offsets scaled by σ, as in prior work [40, 41]. For each mutation step, R=200 perturbed sets were used to locally explore the solution space (Equation 9). ID‐MC was evaluated with $N_{m}=1$ to 10. Source code will be available at https://github.com/dan‐benjamini/mdmri‐idmc.

Voxel‐wise parameter distributions were derived from bootstrap estimates. For each voxel, sets were aggregated into multidimensional distributions and projected onto scalar metrics ($D_{\text{iso}}(\omega )$ and $D_{\Delta }^{2}(\omega )$) across ω=6.6–21 Hz [65]. We computed voxel‐wise means E[x], variances V[x], and covariances C[x,y], including within sub‐regions (“bins”) in parameter space. Previous studies have defined three bins in the in vivo human brain, roughly reflecting microstructural profiles of WM (anisotropic, low diffusivity), GM (isotropic, low diffusivity), and CSF (isotropic, high diffusivity) [41]. These in vivo bins, which will be, respectively, referred to as bin 1, 2, and 3, represent partial integration regions in the $D_{\text{iso}}$–$D_{\Delta }^{2}$ distribution space, i.e., bin 1: $D_{\text{iso}}<$ 2.5 μm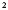/ms and $D_{\Delta }^{2}>$0.25; bin 2: $D_{\text{iso}}<$ 2.5 μm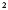/ms and $D_{\Delta }^{2}<$0.25; and bin 3: $D_{\text{iso}}>$ 2.5 μm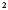/ms and the full range of $D_{\Delta }^{2}$. The normalized weights of these bins were mapped and are labeled as $f_{bin1}$, $f_{bin2}$, and $f_{bin3}$.

To characterize diffusion frequency dependence, we computed finite‐difference estimates of frequency‐related changes in diffusivity metrics across 6.6–21 Hz, as in previous oscillating gradient studies [66]. These included $\Delta_{\omega /2\pi }E[x],\Delta_{\omega /2\pi }V[x]$, and $\Delta_{\omega /2\pi }C[x,y]$.

### 3.4 Reproducibility Analysis

To assess test‐retest reliability and repeatability of the derived parameter maps, intraclass correlation coefficients (ICC) [67], and within‐subject coefficient of variation ($CV_{ws}$) [68] were computed at both voxel‐wise and ROI levels, following prior work [45].

Voxel‐wise analysis enabled fine‐grained mapping by aligning each subject's parameter maps to a study‐specific template using Advanced Normalization Tools (ANTs) [69] and high‐resolution T
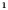
‐weighted images, with session‐wise registration to minimize bias [70]. Spatial Gaussian smoothing (FWHM = 4 mm) was applied to reduce residual misalignment and noise. Complementarily, ROI‐based analysis was performed using SLANT [71] for GM and the Johns Hopkins DTI‐based atlas (https://identifiers.org/neurovault.collection:264) for WM segmentation. ROIs included subcortical GM (basal ganglia and thalamus), cortical GM (middle occipital gyrus and inferior frontal gyrus), and WM tracts (genu, body, and splenium of the corpus callosum (CC), internal capsule, corona radiata, sagittal stratum, and external capsule). Voxels with overlapping GM/WM labels or high CSF content were excluded. Parameter values were averaged within each ROI to reduce spatial noise and enable hypothesis‐driven interpretation. Full details of spatial normalization and ICC/$CV_{ws}$ computation are provided in our previous work [45].

## 4 Results

### 4.1 Numerical Simulation

**FIGURE 2:**
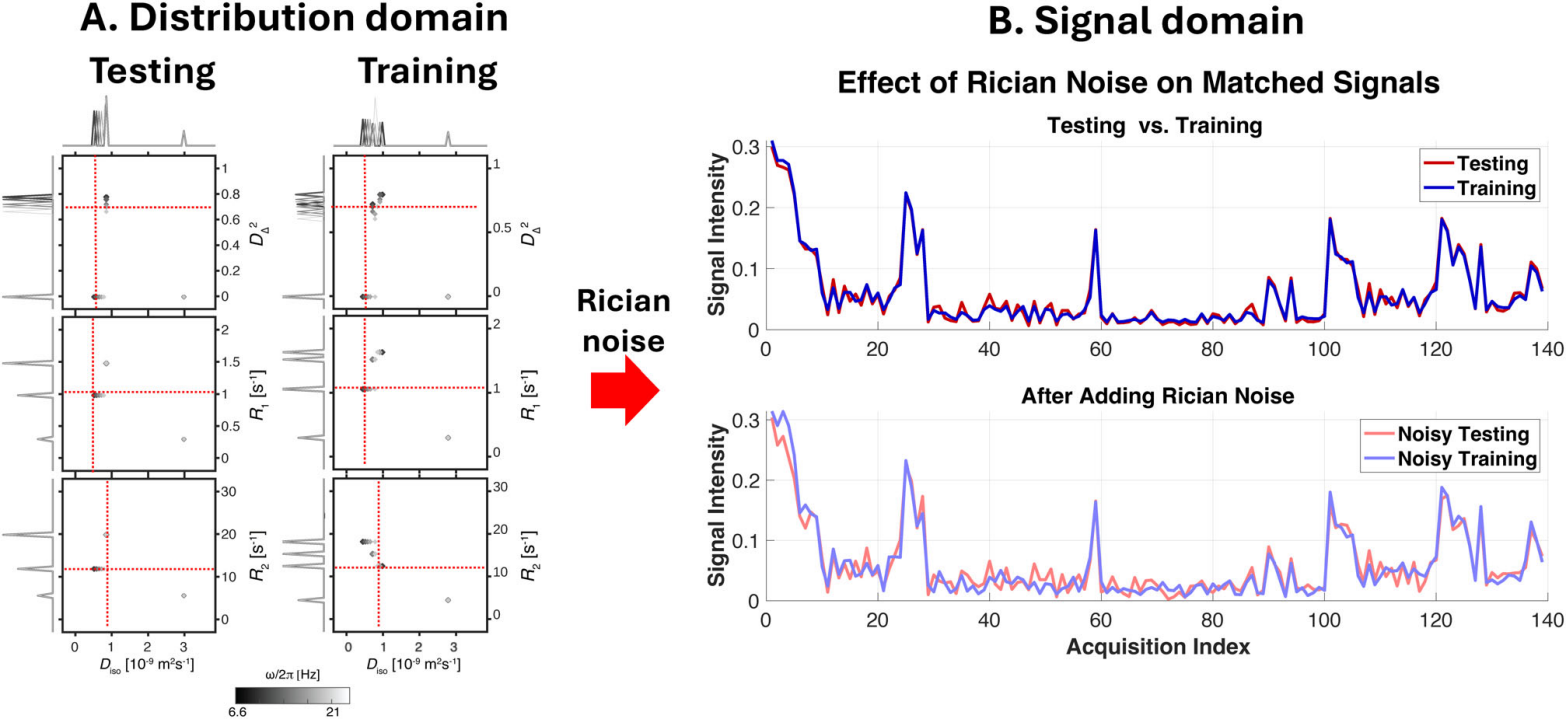
Comparison between testing and training sets ($\sigma_{\text{bias}}=0.2$). (A) 2D projections of the testing and training. (B) Comparison between testing signal and the training signal before and after the addition of Rician noise. The top row illustrates training and testing signals in the noise‐free condition. The bottom row shows the same signals after Rician noise is added, simulating realistic measurement conditions. Note that although the testing and training signals are quite similar in the noise‐free condition, the addition of noise causes the patterns to diverge, effectively resulting in distinct signals.

Figure 2 shows the ground‐truth (testing) and training distributions (with $\sigma_{\text{bias}}=0.2$) and compares the corresponding signals before and after adding Rician noise. Under noise‐free conditions (Figure 2B, top row), the two signal sets exhibit similar patterns. However, even when such bias is small, or when measurement noise is introduced (testing SNR = 130; training SNR = 30) to emulate realistic acquisition conditions, noticeable visual differences emerge between the two signal sets (Figure 2B, bottom row). Although the underlying D(ω)–$R_{1}$–$R_{2}$ distributions remain similar, this demonstrates how measurement noise can obscure true similarity, leading to underfitting and bias in parameter estimation, and ultimately reducing inversion accuracy.

**FIGURE 3:**
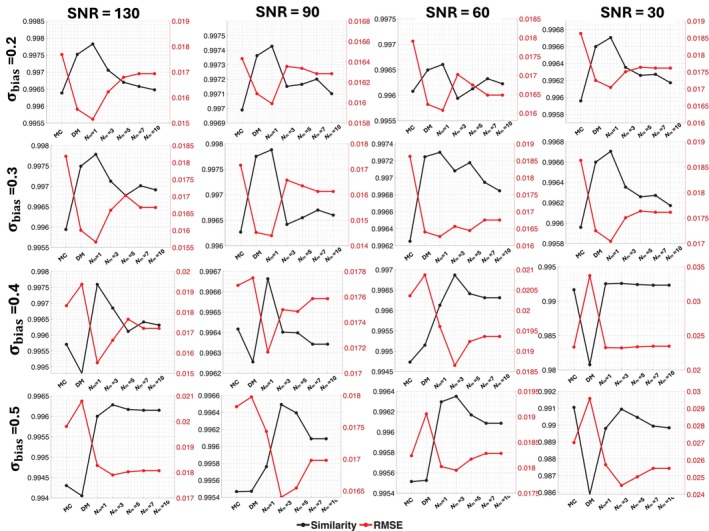
Comparison of similarity and RMSE using MC, DM, and ID‐MC for four SNR levels (130, 90, 60, 30) and four dictionary bias levels (0.2, 0.3, 0.4, 0.5). Each subplot shows the mean similarity (black) and RMSE (red) averaged over all voxels within a given SNR and $\sigma_{\text{bias}}$. Notably, ID‐MC with a low mutation level ($N_{m}=1$) improves similarity and reduces RMSE compared to DM. However, increasing the mutation level ($N_{m}=3$ to 10) leads to reduced similarity and increased RMSE, indicating reduced overall fitting performance. It is noted that a small amount of mutation effectively alleviates underfitting and enhances fitting accuracy, whereas large mutation level may lead to overfitting, reducing generalization performance.

Figure 3 shows the mean similarity (black) and root‐mean‐square error (RMSE, red) between reconstructed and testing signals across four SNR levels with four dictionary bias levels. Each subplot summarizes the average results from 10,000 simulated voxels per SNR and bias condition. Across all SNR and bias levels under $\sigma_{\text{bias}}=0.5$, ID‐MC with a single mutation step ($N_{m}=1$) consistently achieves the highest similarity and lowest RMSE, demonstrating superior estimation accuracy and robustness to moderate prior deviations. At higher mutation levels ($N_{m}>3$), similarity gradually decreases and RMSE increases, likely due to noise overfitting. These results emphasize that ID‐MC maintains stable performance across a wide range of dictionary biases, underscoring the importance of selecting an optimal mutation depth to balance accuracy and reproducibility.

**FIGURE 4:**
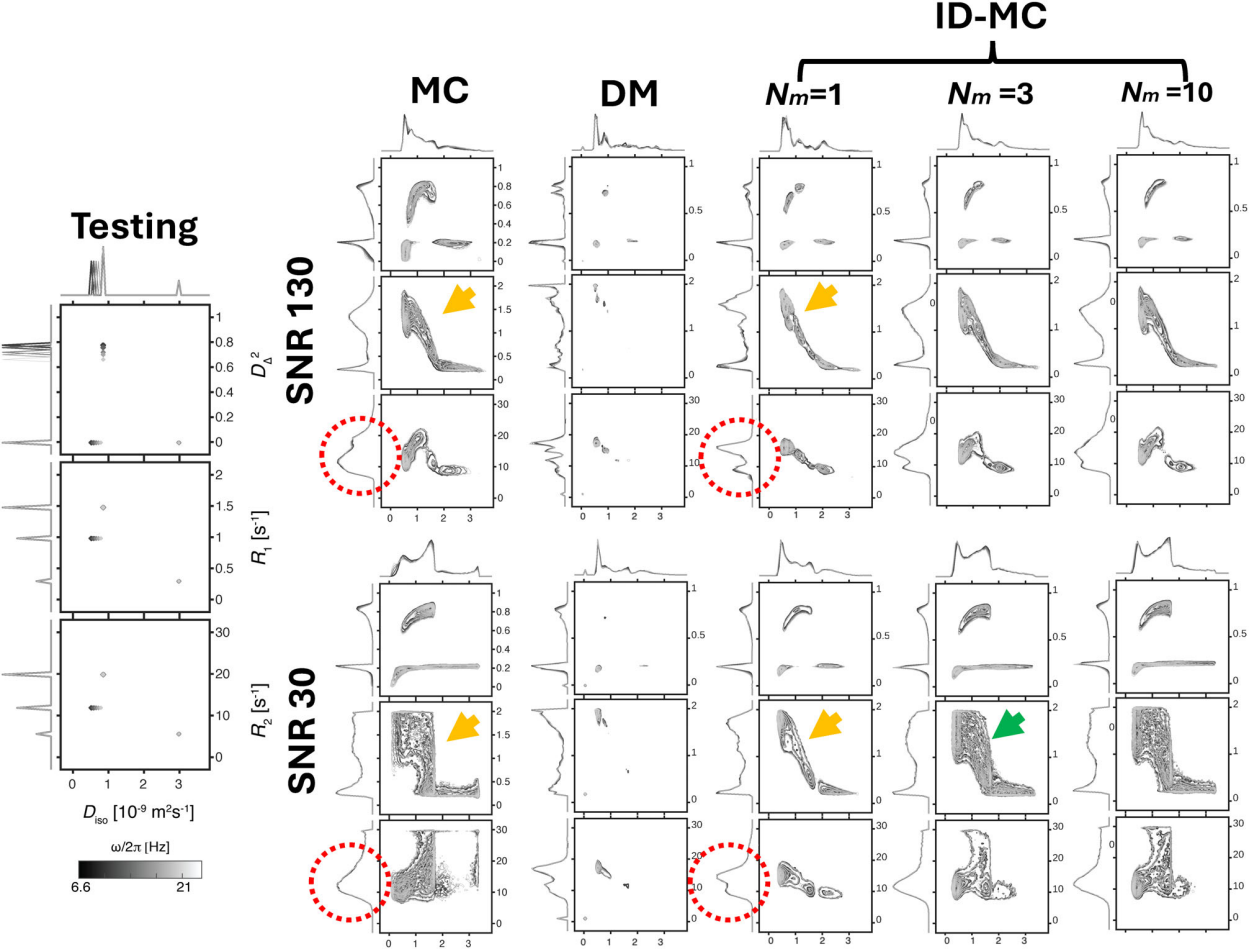
Comparison of 2D projections of the estimated D(ω)–$R_{1}$–$R_{2}$ distributions across methods ($\sigma_{\text{bias}}=0.2$). Projections include $D_{\text{iso}}$–$D_{\Delta }^{2}$ (top), $D_{\text{iso}}$–$R_{1}$ (middle), and $D_{\text{iso}}$–$R_{2}$ (bottom). DM and ID‐MC show narrower distributions, indicating improved estimation stability (orange arrows). At higher mutation level ($N_{m}>3$), the distributions become broader, suggesting potential overfitting (green arrow). ID‐MC also demonstrates more distinct spectral components, reflecting better resolution of heterogeneous tissue environments (red dotted circle).

Figure 4 shows 2D projections of estimated $D(\omega )-R_{1}-R_{2}$ distributions across reconstruction methods (with $\sigma_{\text{bias}}=0.2$). Projections include $D_{\text{iso}}-D_{\Delta }^{2}$ (top), $D_{\text{iso}}-R_{1}$ (middle), and $D_{\text{iso}}-R_{2}$ (bottom) at five diffusion frequencies ω/2π from 6.6 to 21 Hz, evaluated for varying mutation levels ($N_{m}=1,3,10$) in ID‐MC, with MC and DM as references. Both DM and ID‐MC yield narrower, more localized distributions than MC (orange arrows), suggesting better stability and precision. ID‐MC also shows clearer spectral delineation (red dotted circle), reflecting enhanced sensitivity to microstructural heterogeneity. This benefit is most prominent at a moderate mutation level ($N_{m}=1$), balancing perturbation and regularization for accurate inference. Beyond $N_{m}>3$, distributions grow broader and less defined (green arrow), indicating reduced fidelity likely due to noise overfitting. These parameter domain findings are in agreement with the signal domain results shown in Figure 3.

**FIGURE 5:**
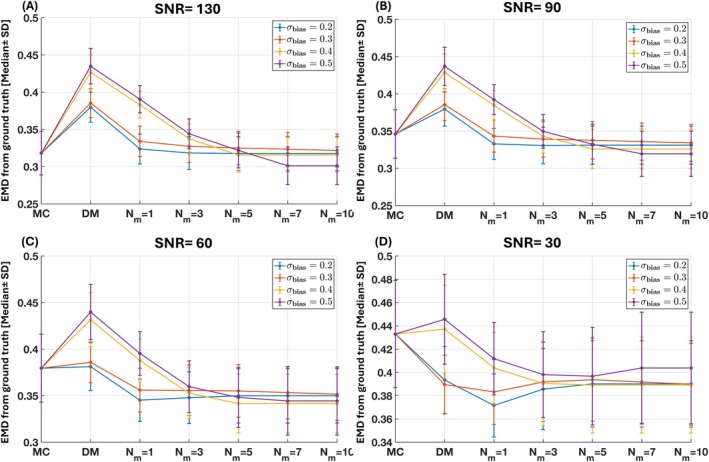
Comparison of mutation effects under varying dictionary bias and SNR levels. Median ± SD Earth Mover's Distance (EMD) is shown for MC, DM, and ID‐MC across four dictionary bias levels (Bias 1–4; $\lambda_{\text{bias}}=[0.2,0.3,0.4,0.5]$) and SNRs of 130, 90, 60, and 30. Each bias level represents increasing deviation of the dictionary prior from the ground‐truth distribution. When the prior is well aligned with the true signal space, all methods perform comparably; however, as the prior becomes more biased or the SNR decreases, the mutation process in ID‐MC becomes increasingly beneficial, yielding lower median EMD and reduced variability.

Figure 5 illustrates the impact of dictionary bias and mutation level on reconstruction performance across SNRs, showing the median and standard deviation of voxel‐wise EMD between estimated and ground‐truth distributions. Across all conditions, ID‐MC consistently achieves lower EMD values than standard MC, indicating higher accuracy. At moderate‐to‐low bias levels ($\lambda_{\text{bias}}<0.4$), ID‐MC with one mutation step ($N_{m}=1$) yields the lowest median EMD and variability, providing the most stable reconstructions. Under low‐SNR and high‐bias conditions (Figure 5D), EMD rises when $N_{m}>3$, likely due to noise amplification and overfitting, whereas at higher SNRs (Figure 5A,B), mutation‐induced variability plateaus as noise effects diminish. Overall, ID‐MC remains robust for moderate mutation levels ($N_{m}\leq 1$–3) under noisy and biased conditions. The increase in EMD with stronger bias is most evident at low SNRs, reflecting compounding effects of noise and prior mismatch. L‐curve analysis identified $N_{m}=1$ as the optimal trade‐off between accuracy, stability, and efficiency.

For completeness, we also show the percent difference between ground truth and simulation values of the different MD‐MRI mean and variance parameters, for all levels of SNR and dictionary bias (Figures S2–S5).

### 4.2 In Vivo Evaluation

**FIGURE 6:**
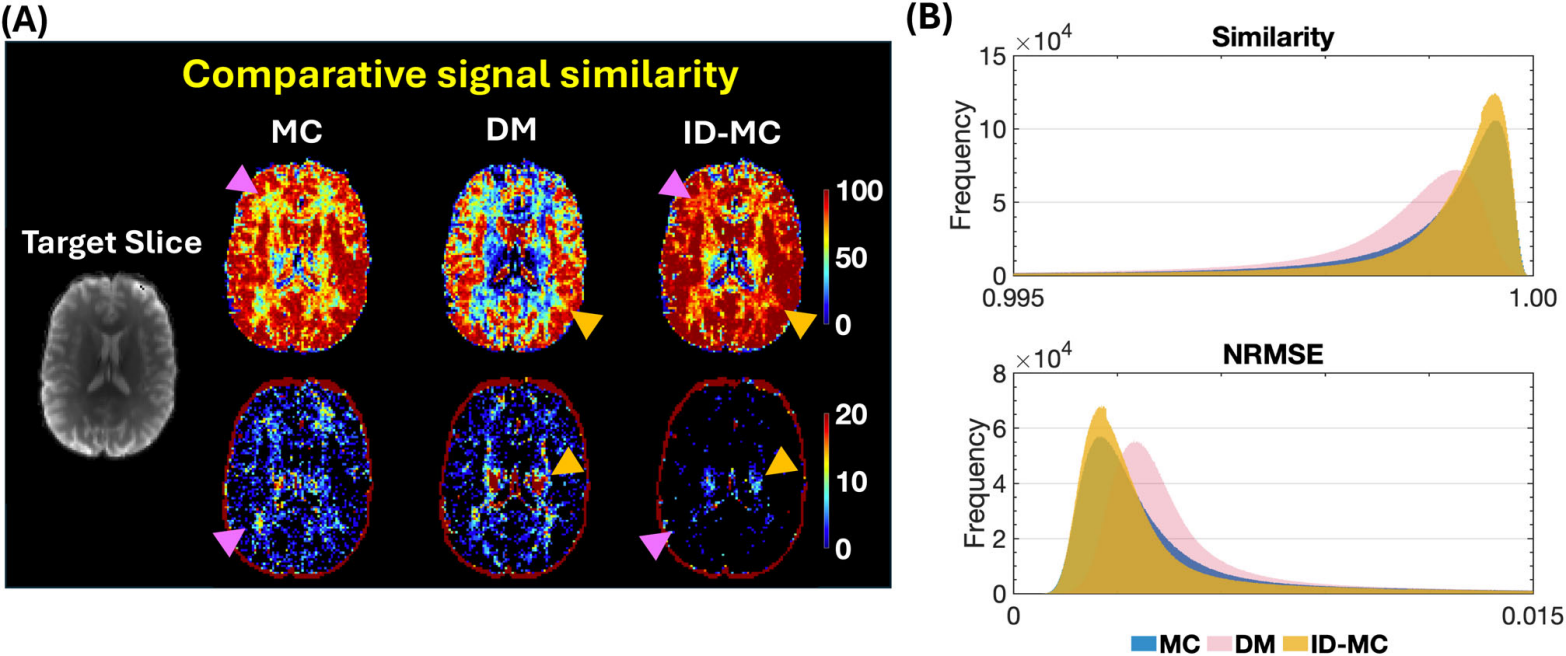
In vivo fitting accuracy. (A) In a representative slice, the top row shows the number of bootstrap signals (out of $N_{b}=100$) that exhibit high similarity (>0.999) with each bootstrap signal at each voxel, while the bottom row shows the count of signals with low similarity (<0.997). ID‐MC demonstrates improved consistency compared to MC (pink arrows) and highlights the impact of mutation when compared to DM (orange arrows). (B) Histograms show the distributions of similarity and NRMSE across all voxels in the corresponding slice of the same subject from (A). ID‐MC reduces underfitting, as shown by a shift toward higher similarity and lower error.

Figure 6 evaluates ID‐MC fitting accuracy ($N_{m}=1$) using signal similarity. For a representative slice, similarity among 100 bootstrap signals per voxel was computed. In Figure 6A, the top row shows the number of bootstrap signals exceeding a similarity threshold of 0.999, while the bottom row displays those falling below 0.997. These thresholds correspond to the 80^th^ and 20^th^ percentiles of the similarity distribution in the training data set. Compared to MC, ID‐MC yields more high‐similarity signals (pink arrows) and fewer low‐similarity ones than DM (orange arrows), indicating improved stability and reduced underfitting. Notably, the largest improvements were observed in WM regions with crossing fibers, where conventional MC inversion often becomes unstable due to parameter degeneracy and multiple local optima. By constraining the inversion within a biophysically plausible dictionary subspace and allowing localized mutation‐based refinement, ID‐MC achieves more stable reconstruction of heterogeneous fiber configurations. Figure 6B shows similarity and normalized RMSE histograms, with ID‐MC clearly shifted toward higher similarity and lower error, confirming improved accuracy and reduced bias.

**FIGURE 7:**
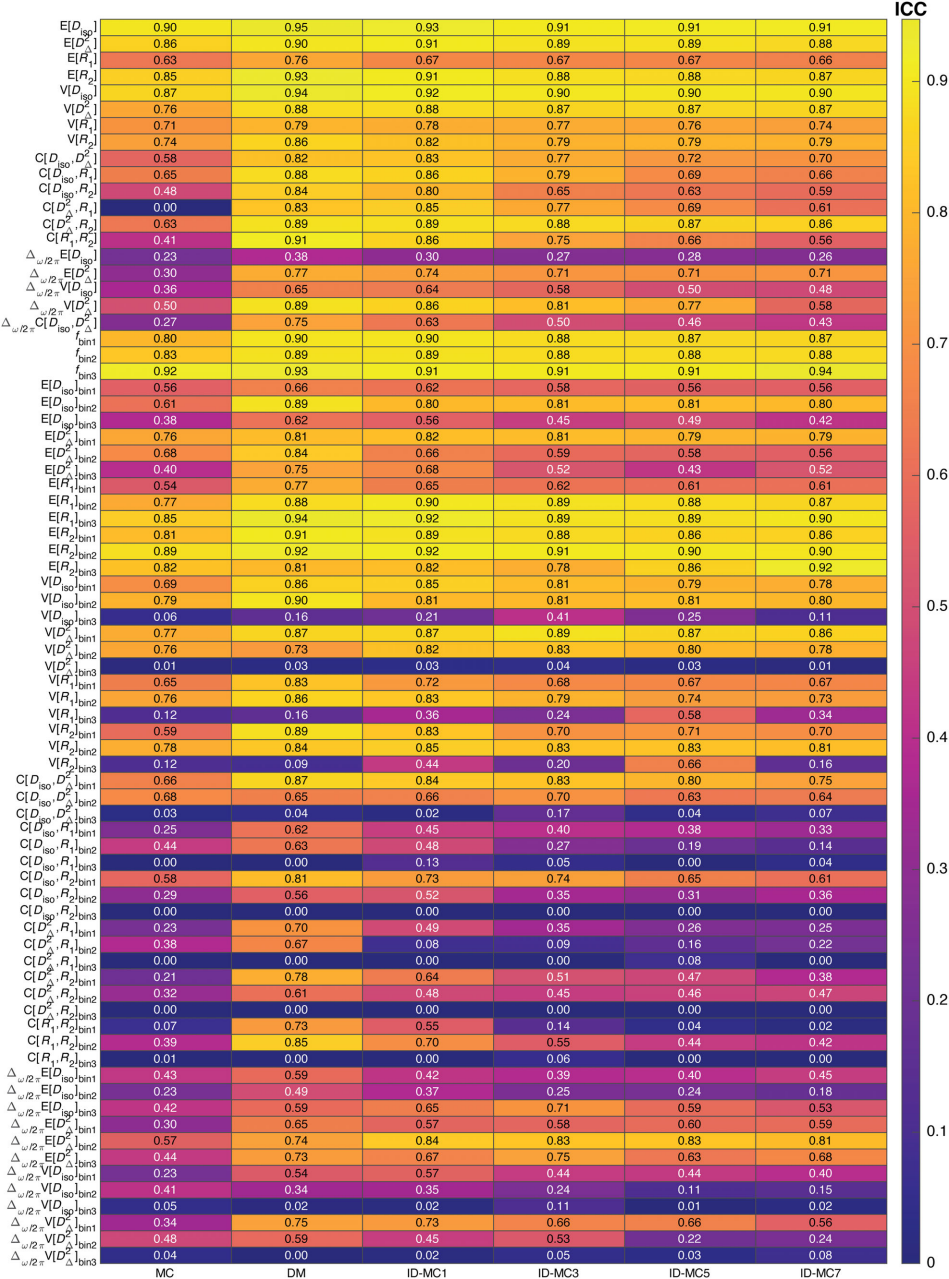
Intraclass Correlation Coefficient (ICC) heatmap of ROI‐based parameter estimates across different inversion methods. Each row corresponds to an MD‐MRI parameter, while each column represents an inversion strategy: MC, DM, and ID‐MC with different mutation levels ($N_{m}=1$ to $N_{m}=7$). The color scale reflects ICC values, with yellow indicating higher reproducibility and purple indicating lower reproducibility. Negative ICC values were zeroed.

Although fitting accuracy is informative, it does not ensure reliable parameter estimates. Because ground truth $D(\omega )-R_{1}-R_{2}$ distributions are unavailable in vivo, we assessed test–retest reproducibility as a practical reliability measure. Following our prior MD‐MRI study [45], we analyzed the same cohort and computed median ICCs across ROIs for each parameter and inversion method. Figure 7 shows a heatmap summarizing these results, with rows denoting parameters (e.g., $E[D_{\text{iso}}],E[R_{2}],V[D_{\Delta }^{2}]$) and columns representing MC, DM, and ID‐MC with $N_{m}=1$–7. ID‐MC at $N_{m}=1$ consistently improved ROI‐level reproducibility compared with MC, particularly for frequency‐dependent parameters such as $\Delta_{\omega /2\pi }E[x]$ and $\Delta_{\omega /2\pi }V[x]$. ICCs generally peaked at low $N_{m}$ and declined at higher values, suggesting limited mutation enhances stability without amplifying noise. Similar trends are observed for the voxelwise ICC, ROI‐based $CV_{ws}$, and voxelwise $CV_{ws}$, which are shown in Figures S6–S8, respectively.

Among the 76 MD‐MRI parameters, those with ICC >0.75 (i.e., good or excellent reliability [72]) included 19 for MC, 41 for DM, and 33, 31, 26, and 23 for ID‐MC with $N_{m}=1$, 3, 5, and 7, respectively. For example, ICCs for $\Delta_{\omega /2\pi }E[D_{\Delta }^{2}]$ and $\Delta_{\omega /2\pi }V[D_{\Delta }^{2}]$ improved from 0.30 and 0.50 (MC) to 0.74 and 0.86 (ID‐MC at $N_{m}=1$), respectively, while ROI $CV_{\text{ws}}$ for $\Delta_{\omega /2\pi }E[D_{\text{iso}}]$ dropped from 1.03 (MC) to 0.13 (ID‐MC; Figure S7). Similar improvements were observed for bin‐resolved frequency‐dependent parameters.

**FIGURE 8:**
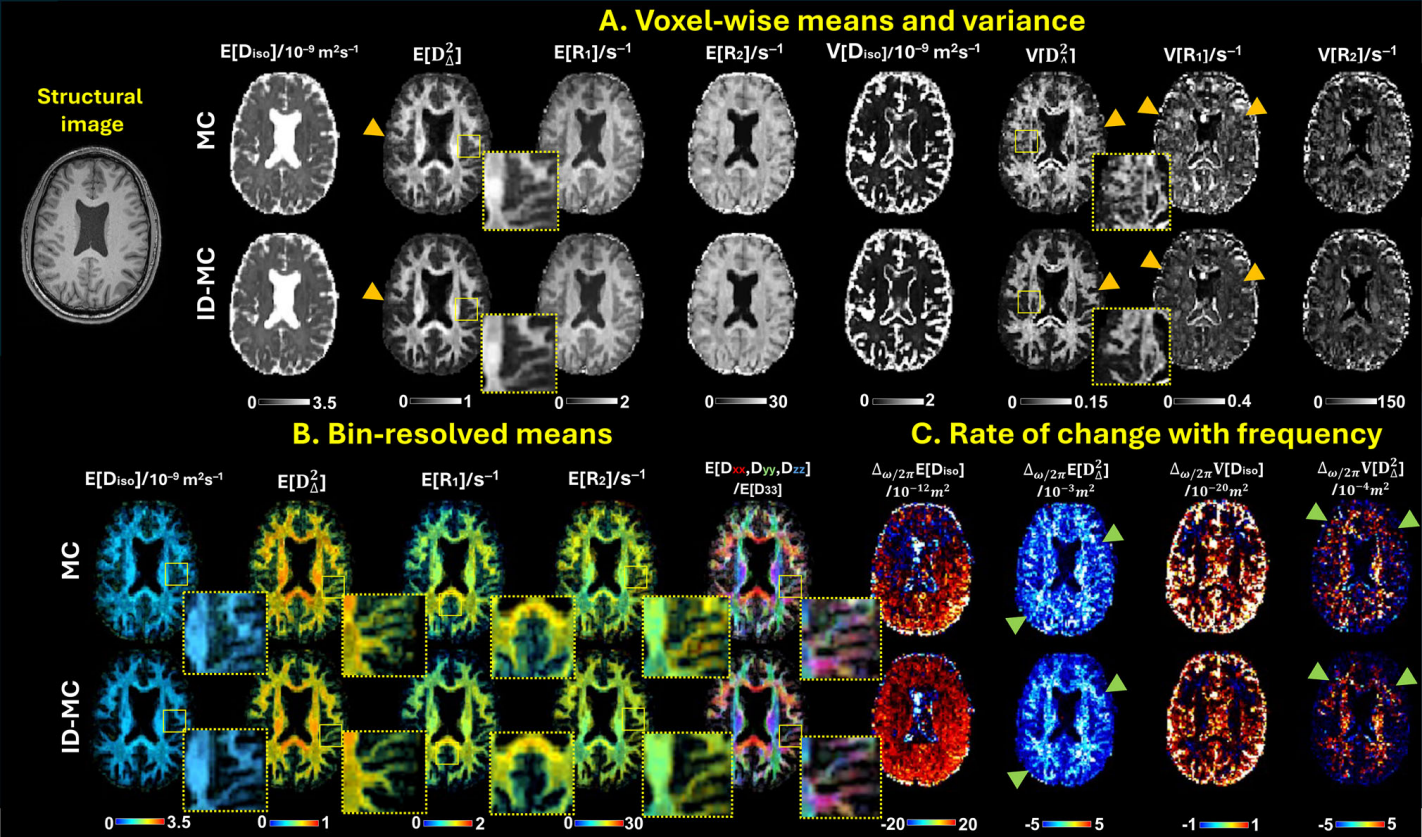
Qualitative comparison of MD‐MRI parameter maps generated by MC and ID‐MC methods. ID‐MC (with $N_{m}=1$) yields superior noise reduction (orange arrows) and clearer structural definition (zoomed images) across various parameters. Furthermore, the rate of change in parameter means and variances as a function of diffusion encoding frequency reveals not only clearer structural delineation but also a substantial reduction in apparent noise (green arrows).

Figure 8 presents the qualitative evaluation of mutation‐based refinement in parameter map estimation. Representative maps from MC and ID‐MC ($N_{m}=1$) were selected based on prior reproducibility findings [45]. ID‐MC produces visibly cleaner maps with reduced noise (orange arrows) and sharper structural definition, particularly in WM regions. These improvements are consistent across parameters sensitive to microstructural heterogeneity and relaxation dynamics. Analysis of parameter means and variances versus diffusion encoding frequency ($\Delta_{\omega /2\pi }$E[x], $\Delta_{\omega /2\pi }$V[x]) further shows that ID‐MC generates smoother, less noisy maps and enhances structural contrast, with frequency‐related trends reflecting improved signal quality and reduced apparent noise.

For completeness, we provide in Figures S9–S11 the estimated MD‐MRI parameter values by all methods in all the investigated ROIs, and compare them to previously published results [45].

Table 2 summarizes the computational time per bootstrap for each inversion method. Compared to MC inversion, which required 13.6 min per bootstrap, DM and ID‐MC reduced computation time to 3.2 and 4.2 min, respectively. Notably, ID‐MC achieved this improvement with only a modest increase over DM while incorporating mutation‐based refinement to enhance parameter estimation robustness.

**TABLE 2:** Computational time per bootstrap.

Method	MC	DM	ID‐MC^a^
Time (min)^b^	13.6±0.3	3.2±0.2	4.2±0.3
Relative Change (%)^c^		−76	−69

^a^ Computation performed with mutation level $N_{m}=1$.

^b^ Hardware: AMD EPYC 7773X 64‐Core Processor, 2 TB RAM.

^c^ With respect to MC inversion.

## 5 Discussion

This work presents a robust and efficient framework for estimating multidimensional joint distributions of frequency‐dependent diffusion and relaxation parameters in brain tissue using an ID‐MC approach. By combining data‐driven, kernel‐subspace‐informed dictionary matching with targeted local mutation, the method improves accuracy, reproducibility, and convergence efficiency compared to MC inversion, while mitigating the underfitting risk associated with pure dictionary‐based inversion (DM).

The state‐of‐the‐art MD‐MRI inversion, the MC pipeline [9, 41, 43], explores high‐dimensional parameter space through stochastic proliferation followed by local mutation. While flexible and compatible with complex models such as our $D(\omega )-R_{1}-R_{2}$ formulation‐it suffers from high computational cost (Table 2) and instability at low SNR due to inefficient sampling of redundant or low‐likelihood parameter regions, which amplifies noise and reduces reproducibility.

We present a computational framework that enhances stochastic global exploration by integrating prior knowledge linking similarity in the kernel subspace to proximity in parameter space. An informed dictionary derived from training data provides biologically plausible signal–parameter pairs, allowing each bootstrap signal to be efficiently matched to its closest entry via cosine similarity. This targeted search greatly reduces computation time by avoiding exhaustive random sampling. Conceptually, the ID‐MC framework bridges dictionary‐ and Monte Carlo–based approaches: unlike model‐constrained methods such as AMICO [73], which rely on fixed biophysical models (e.g., NODDI), ID‐MC remains model‐free and data‐driven, reconstructing full multidimensional parameter distributions without compartmental assumptions. While pure dictionary inversion (i.e., DM) can underfit signals that deviate from predefined entries due to noise or biological variability, ID‐MC addresses this by introducing a local mutation step that perturbs the matched parameters. This refinement enables exploration of nearby configurations that better capture signal features, achieving a balance between local adaptability and global plausibility.

To evaluate the performance of MC, DM, and ID‐MC, we conducted in silico simulations under biologically realistic conditions across varying SNRs and assessed in vivo test–retest reproducibility of MD‐MRI parameters in the human brain. Simulated signals were generated from a voxel with four water pools: two orthogonal fiber populations, an isotropic low‐diffusivity component, and free water (Table 1, Figure 4). At a relatively high SNR of 130, MC outperformed DM, producing estimates closer to the ground truth (Figure 5). Adding mutation to DM (ID‐MC) improved flexibility and surpassed MC in accuracy. At lower SNRs (<90), MC yielded the least accurate estimates, likely from noise overfitting–an effect exacerbated by higher mutation levels, as indicated by increased EMD (Figure 5). In contrast, low mutation levels (e.g., $N_{m}=1$) mitigated underfitting by modestly broadening the solution space to better account for measurement noise and biological variability, yielding improved similarity, lower RMSE, and reduced EMD. Beyond $N_{m}=3$, performance declined, with broader, less distinct distributions and higher EMD, reflecting a shift from underfitting to overfitting. This trade‐off is typical of inverse problems, where greater flexibility improves adaptability but also heightens the risk of fitting noise.

Beyond improvements in EMD and RMSE, the informed dictionary in ID‐MC yields substantial gains in reproducibility in vivo. Test‐retest experiments in a human cohort showed that ID‐MC improves both voxel‐wise and ROI‐based reliability of key diffusion‐relaxation parameters, as indicated by higher ICC and lower $CV_{\text{ws}}$ compared to MC (Figures 7 and S6–S8). ID‐MC doubled the number of MD‐MRI parameters achieving ICC values above 0.75–indicative of good or excellent reliability. This was especially notable for diffusion frequency‐dependent parameters, which showed poor reproducibility with MC [45]. With ID‐MC, these parameters achieved up to a 146% increase in ICC and an 87% reduction in $CV_{ws}$, underscoring its potential for more reliable and biologically meaningful assessments.

Although DM inversion yields the greatest reproducibility improvement over MC, our in silico results reveal limitations of relying solely on a dictionary‐based approach and highlight the need for careful mutation tuning. Based on both in silico and in vivo findings, we suggest that ID‐MC with a single mutation step ($N_{m}=1$) provides a good balance between stability and flexibility, enabling accurate recovery of multidimensional parameter distributions without overfitting. The optimal mutation level may vary depending on the application and training set characteristics; for instance, cohorts with high pathological heterogeneity may benefit from more extensive mutation tuning.

In addition to gains in accuracy and in vivo test retest reproducibility, the informed dictionary‐based inversion approach offers a major advantage in computational efficiency. As shown in Table 2, average computation time per bootstrap dropped from about 13.6 min with standard MC inversion to 4.2 min using ID‐MC ($N_{m}=1$), a more than threefold improvement. This level of efficiency is especially important for translating MD‐MRI into practical use, where whole‐brain, multivoxel, multisubject processing is required.

While the ID‐MC framework improves efficiency and stability, several limitations remain. The method currently relies on a data‐driven dictionary built from normative brain data acquired at a single site, without synthetic augmentation or multisite variability, which may limit generalizability to pathology or different MRI systems. If disease‐specific microstructural patterns are not represented, the framework may fail to capture such abnormalities even when they fall within the theoretical parameter space, underscoring the need to expand the dictionary to include pathological, multisite, or simulated examples. Estimating SNR in vivo also presents challenges due to variability in subjects, hardware, physiological noise, and tissue heterogeneity, complicating robustness assessments across noise levels. The mutation level ($N_{m}$) was selected empirically, and incorporating automated tuning strategies, such as cross‐validation or L‐curve optimization, may enhance reproducibility. Although the current MATLAB implementation provides meaningful acceleration, further gains could be achieved through GPU parallelization or the integration of sequential Monte Carlo approaches to improve stability and convergence. Finally, while this work focused on methodological validation, future studies should demonstrate the clinical utility of ID‐MC–derived parameters in applications such as axonal injury, neurodegeneration, neuroinflammation, and diffusion–relaxation decoupling.

## 6 Conclusion

The methodological advances presented here have important implications for expanding the utility of MD‐MRI in clinical and translational neuroscience. Brain tissue exhibits a complex mixture of microenvironments, from densely packed anisotropic myelinated fibers in WM, to more isotropic cellular structures in GM. Each of these features contributes distinct frequency‐dependent diffusion‐relaxation signatures, which collectively reflect the underlying microstructural architecture. The ability of the proposed ID‐MC framework to robustly resolve these multidimensional characteristics across repeated scans suggests that it can serve as a stable foundation for identifying and monitoring subtle microstructural changes in the brain. This capability is particularly relevant to studies of Alzheimer's disease and dementia [14] or traumatic brain injury [28], where early or progressive alterations in tissue microstructure may manifest as modest shifts in diffusion‐relaxation spectra that would otherwise be obscured by noise or inversion instability.

## Funding

This research was supported by the Intramural Research Program of the National Institutes of Health (NIH). The contributions of the NIH author(s) are considered Works of the United States Government. The findings and conclusions presented in this paper are those of the author(s) and do not necessarily reflect the views of the NIH or the US Department of Health and Human Services.

## Conflicts of Interest

The authors declare no conflicts of interest.

## Supporting information




**Data S1: Figure S1:** Key experimental details. (A) Time‐dependent effective gradients G(t) and (B) corresponding tensor‐valued encoding spectra b(ω) for linear, planar, and spherical encoding at different echo times and centroid frequencies, denoted by black vertical lines. (C) Acquisition protocol with repetition time TR, echo time TE, as well as b‐tensor magnitude b, normalized anisotropy $b_{\Delta }$ (planar: 0.5, spherical: 0, linear: 1), orientation (Θ,Φ), and centroid frequency $\omega_{cent}/2\pi$, versus image acquisition index.
**Figure S2:** Percent difference from ground truth of mean E[x] and variance V[x] parameters for the different methods under different dictionary bias conditions at SNR of 130.
**Figure S3:** Percent difference from ground truth of mean E[x] and variance V[x] parameters for the different methods under different dictionary bias conditions at SNR of 90.
**Figure S4:** Percent difference from ground truth of mean E[x] and variance V[x] parameters for the different methods under different dictionary bias conditions at SNR of 60.
**Figure S5:** Percent difference from ground truth of mean E[x] and variance V[x] parameters for the different methods under different dictionary bias conditions at SNR of 30.
**Figure S6:** Intraclass Correlation Coefficient (ICC) heatmap of median voxel‐wise parameter estimates across different inversion methods. Each row corresponds to an MD‐MRI parameter, while each column represents an inversion strategy: MC, DM, and ID‐MC with different mutation levels ($N_{m}=1$ to $N_{m}=7$). The color scale reflects ICC values, with yellow indicating higher reproducibility and purple indicating lower reproducibility. Negative ICC values were zeroed.
**Figure S7:** Within‐subject coefficient of variation ($CV_{ws}$) heatmap of ROI‐based parameter estimates across different inversion methods. Each row corresponds to an MD‐MRI parameter, while each column represents an inversion strategy: MC, DM, and ID‐MC with different mutation levels ($N_{m}=1$ to $N_{m}=7$). The color scale reflects $CV_{ws}$ values, with purple indicating higher reproducibility and yellow indicating lower reproducibility.
**Figure S8:** Within‐subject coefficient of variation ($CV_{ws}$) heatmap of median voxel‐wise parameter estimates across different inversion methods. Each row corresponds to an MD‐MRI parameter, while each column represents an inversion strategy: MC, DM, and ID‐MC with different mutation levels ($N_{m}=1$ to $N_{m}=7$). The color scale reflects $CV_{ws}$ values, with purple indicating higher reproducibility and yellow indicating lower reproducibility.
**Figure S9:** Mean of region‐of‐interest‐averaged MD‐MRI parameters, obtained using the different processing approaches. Abbreviations: MOG, middle occipital gyrus; IFG, inferior frontal gyrus; CC genu, genu of the corpus callosum; CC body, body of the corpus callosum; CC splenium, splenium of the corpus callosum.
**Figure S10:** Mean of region‐of‐interest‐averaged MD‐MRI parameters, obtained using the different processing approaches. Abbreviations: MOG, middle occipital gyrus; IFG, inferior frontal gyrus; CC genu, genu of the corpus callosum; CC body, body of the corpus callosum; CC splenium, splenium of the corpus callosum.
**Figure S11:** Mean of region‐of‐interest‐averaged MD‐MRI parameters, obtained using the different processing approaches. Abbreviations: MOG, middle occipital gyrus; IFG, inferior frontal gyrus; CC genu, genu of the corpus callosum; CC body, body of the corpus callosum; CC splenium, splenium of the corpus callosum.
**Table S1:** Comprehensive summary of the in vivo MD‐MRI acquisition protocol.
**Table S2:** Dictionary simulation components for bias‐controlled experiments.

## Data Availability

The data that support the findings of this study are available on request from the corresponding author. The data are not publicly available due to privacy or ethical restrictions. Code to process the MD‐MRI data using the MC inversion method is freely available as implemented in the multidimensional diffusion MRI toolbox (https://github.com/markus‐nilsson/md‐dmri). Code to process the MD‐MRI data using the ID‐MC inversion method will be available at https://github.com/dan‐benjamini/mdmri‐idmc.
