# Supplementary material for: Informed Dictionary‐Guided Monte Carlo Inversion for Robust and Reproducible Multidimensional MRI
Source: Magn Reson Med. 2025 Dec 28;95(5):2947–62. doi: 10.1002/mrm.70228 (PMC12962224; doi:10.1002/mrm.70228)
Supplement: Supplementary file 1 — Data S1: Figure S1: Key experimental details. (A) Time‐dependent effective gradients G(t) and (B) corresponding tensor‐valued encoding spectra b(ω) for linear, planar, and spherical encoding at different echo times and centroid frequencies, denoted by black vertical lines. (C) Acquisition protocol with repetition time TR, echo time TE, as well as b‐tensor magnitude b, normalized anisotropy bΔ (planar: 0.5, spherical: 0, linear: 1), orientation (Θ,Φ), and centroid frequency ωcent/2π, versus image acquisition index. Figure S2: Percent difference from ground truth of mean E[x] and variance V[x] parameters for the different methods under different dictionary bias conditions at SNR of 130. Figure S3: Percent difference from ground truth of mean E[x] and variance V[x] parameters for the different methods under different dictionary bias conditions at SNR of 90. Figure S4: Percent difference from ground truth of mean E[x] and variance V[x] parameters for the different methods under different dictionary bias conditions at SNR of 60. Figure S5: Percent difference from ground truth of mean E[x] and variance V[x] parameters for the different methods under different dictionary bias conditions at SNR of 30. Figure S6: Intraclass Correlation Coefficient (ICC) heatmap of median voxel‐wise parameter estimates across different inversion methods. Each row corresponds to an MD‐MRI parameter, while each column represents an inversion strategy: MC, DM, and ID‐MC with different mutation levels (Nm=1 to Nm=7). The color scale reflects ICC values, with yellow indicating higher reproducibility and purple indicating lower reproducibility. Negative ICC values were zeroed. Figure S7: Within‐subject coefficient of variation (CVws) heatmap of ROI‐based parameter estimates across different inversion methods. Each row corresponds to an MD‐MRI parameter, while each column represents an inversion strategy: MC, DM, and ID‐MC with different mutation levels (Nm=1 to Nm=7). The color scale reflects CVws [file MRM-95-2947-s001.pdf]

Supplementary Information: Informed Dictionary-Guided Monte Carlo Inversion for Robust and Reproducible Multidimensional MRI

Joon Sik Park[a], Eppu Manninen[a], Yihong Yang[b], Dan Benjamini[a,*]

[a] *Multiscale Imaging and Integrative Biophysics Unit, Laboratory of Behavioral Neuroscience, National Institute on Aging, NIH, Baltimore, MD, USA*
[b] *Neuroimaging Research Branch, National Institute on Drug Abuse, NIH, Baltimore, MD, USA*

Supplementary Figures

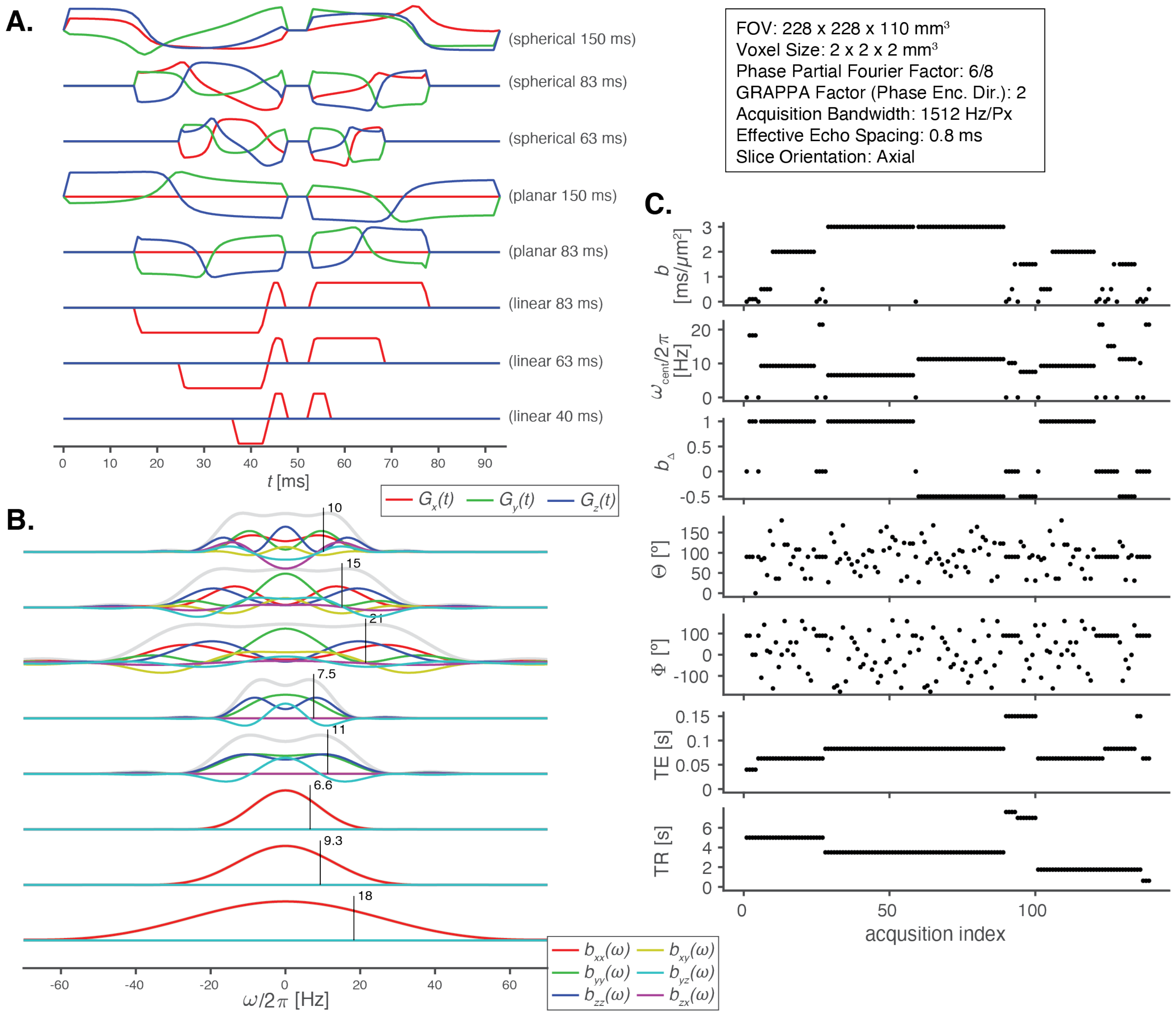

Supplementary Figure S1: Key experimental details. (A) Time-dependent effective gradients $G(t)$ and (B) corresponding tensor-valued encoding spectra $\mathbf{b}(\omega)$ for linear, planar, and spherical encoding at different echo times and centroid frequencies, denoted by black vertical lines. (C) Acquisition protocol with repetition time TR, echo time TE, as well as $\mathbf{b}$-tensor magnitude b, normalized anisotropy b_Δ (planar: –0.5, spherical: 0, linear: 1), orientation (Θ, Φ), and centroid frequency $\omega_{cent}/2\pi$, versus image acquisition index.

*Corresponding author. Phone: +1-667-312-3569
Email address: dan.benjamini@nih.gov (Dan Benjamini)

σ_{bias} = 0.2

% difference from ground truth

	MC	DM	ID-MC1	ID-MC3	ID-MC5	ID-MC7	ID-MC10
$E[D_{iso}]$	10.44	9.82	7.61	6.87	7.51	8.25	8.88
$E[D_{\Delta}^2]$	25.41	9.58	14.13	15.20	16.60	17.70	18.62
$E[R_1]$	4.88	9.97	0.02	3.00	3.68	3.95	4.09
$E[R_2]$	2.44	2.00	0.92	0.47	0.60	1.35	1.82
$V[D_{iso}]$	37.62	24.20	33.86	39.77	40.82	40.87	40.82
$V[D_{\Delta}^2]$	59.06	52.13	51.11	58.65	58.29	57.86	57.46
$V[R_1]$	70.09	101.64	38.11	52.04	44.74	41.05	38.70
$V[R_2]$	14.04	6.22	14.00	7.69	0.46	6.19	9.75
$\Delta_{\omega/2\pi}E[D_{iso}]$	88.89	92.39	52.20	87.87	87.03	86.35	85.88
$\Delta_{\omega/2\pi}E[D_{\Delta}^2]$	51.27	45.97	28.22	54.38	56.14	57.17	57.70
f_{bin1}	34.11	20.06	11.00	21.81	23.63	24.71	25.47
f_{bin2}	27.12	8.10	5.67	5.97	9.38	12.01	14.08
f_{bin3}	80.61	89.48	89.43	91.54	89.93	88.64	87.77

σ_{bias} = 0.3

% difference from ground truth

	MC	DM	ID-MC1	ID-MC3	ID-MC5	ID-MC7	ID-MC10
$E[D_{iso}]$	16.34	30.70	10.44	14.76	14.13	13.82	13.69
$E[D_{\Delta}^2]$	25.41	13.63	17.66	19.13	20.21	20.89	21.51
$E[R_1]$	5.88	7.15	4.88	6.02	5.85	5.74	5.65
$E[R_2]$	2.44	8.40	2.17	0.40	0.67	1.31	1.86
$V[D_{iso}]$	37.62	37.84	12.18	20.11	24.18	26.91	29.76
$V[D_{\Delta}^2]$	51.11	47.44	46.08	46.18	46.24	46.31	46.13
$V[R_1]$	38.11	119.59	56.75	46.69	41.62	38.74	36.38
$V[R_2]$	14.04	39.36	22.73	9.60	1.20	4.27	9.09
$\Delta_{\omega/2\pi}E[D_{iso}]$	103.33	105.98	52.20	101.64	100.43	99.52	98.36
$\Delta_{\omega/2\pi}E[D_{\Delta}^2]$	56.70	49.54	28.22	58.61	59.98	60.74	61.61
f_{bin1}	34.11	11.22	19.26	20.30	20.88	21.13	21.25
f_{bin2}	27.12	16.08	12.75	10.87	10.52	10.50	10.99
f_{bin3}	80.61	11.97	37.84	52.35	59.62	64.14	68.17

σ_{bias} = 0.4

% difference from ground truth

	MC	DM	ID-MC1	ID-MC3	ID-MC5	ID-MC7	ID-MC10
$E[D_{iso}]$	10.44	34.21	11.18	9.24	8.73	8.88	9.52
$E[D_{\Delta}^2]$	25.41	34.37	5.69	28.51	26.20	24.87	23.91
$E[R_1]$	4.88	39.14	13.99	10.01	7.99	6.69	5.66
$E[R_2]$	2.44	10.48	1.88	0.21	0.54	1.09	1.64
$V[D_{iso}]$	37.62	52.10	22.29	49.77	46.46	42.44	38.08
$V[D_{\Delta}^2]$	51.11	62.25	56.13	55.27	55.77	55.84	55.47
$V[R_1]$	38.11	21.79	1.44	36.80	40.81	42.51	42.37
$V[R_2]$	14.04	34.85	31.18	14.77	5.59	0.92	6.66
$\Delta_{\omega/2\pi}E[D_{iso}]$	52.20	56.53	35.11	68.20	68.33	67.97	67.39
$\Delta_{\omega/2\pi}E[D_{\Delta}^2]$	28.22	22.91	20.67	22.70	26.45	29.35	32.43
f_{bin1}	34.11	37.88	0.02	31.28	30.58	29.94	29.36
f_{bin2}	27.12	47.17	35.48	22.18	22.18	22.84	23.53
f_{bin3}	80.61	97.32	61.29	95.67	92.41	86.84	79.00

σ_{bias} = 0.5

% difference from ground truth

	MC	DM	ID-MC1	ID-MC3	ID-MC5	ID-MC7	ID-MC10
$E[D_{iso}]$	10.44	9.39	7.68	5.20	3.70	2.78	2.21
$E[D_{\Delta}^2]$	52.18	59.47	25.41	51.89	49.88	48.47	47.12
$E[R_1]$	4.88	10.30	0.33	10.62	8.00	5.87	3.71
$E[R_2]$	2.44	14.17	9.12	3.51	1.63	0.06	1.76
$V[D_{iso}]$	37.62	25.98	38.45	17.11	20.15	21.41	22.05
$V[D_{\Delta}^2]$	94.31	95.13	51.11	97.35	97.60	97.80	98.15
$V[R_1]$	38.11	6.82	26.16	6.56	6.71	17.61	29.20
$V[R_2]$	14.04	61.78	74.71	47.99	29.96	13.90	2.40
$\Delta_{\omega/2\pi}E[D_{iso}]$	83.30	87.42	52.20	87.00	86.45	86.30	86.25
$\Delta_{\omega/2\pi}E[D_{\Delta}^2]$	78.07	82.23	28.22	82.34	81.02	79.91	78.96
f_{bin1}	100.00	100.00	34.11	100.00	100.00	100.00	100.00
f_{bin2}	115.26	126.53	27.12	127.91	128.17	128.44	129.26
f_{bin3}	80.61	93.31	62.68	66.37	72.45	75.81	77.33

Supplementary Figure S2: Percent difference from ground truth of mean E[x] and variance V[x] parameters for the different methods under different dictionary bias conditions at SNR of 130.

$\sigma_{bias} = 0.2$

% difference from ground truth

	MC	DM	ID-MC1	ID-MC3	ID-MC5	ID-MC7	ID-MC10
$E[D_{iso}]$	12.35	9.90	8.27	7.74	8.40	9.14	9.79
$E[D_\Delta^2]$	30.77	9.49	16.04	17.55	19.18	20.54	21.67
$E[R_1]$	4.62	10.02	0.14	2.79	3.46	3.71	3.82
$E[R_2]$	3.48	1.95	0.66	0.01	1.16	2.00	2.56
$V[D_{iso}]$	40.81	23.92	34.27	40.89	42.70	43.29	43.53
$V[D_\Delta^2]$	54.35	52.40	58.47	58.21	58.07	57.84	57.67
$V[R_1]$	47.49	102.04	71.35	54.95	48.53	45.41	43.45
$V[R_2]$	16.23	4.57	11.96	5.30	3.12	9.14	13.19
$\Delta_{\omega/2\pi}E[D_{iso}]$	90.69	92.51	72.46	90.22	89.86	89.63	89.50
$\Delta_{\omega/2\pi}E[D_\Delta^2]$	48.79	45.91	27.63	51.48	52.92	53.77	54.33
f_{bin1}	35.99	21.70	11.73	23.31	24.97	26.26	27.16
f_{bin2}	30.08	8.37	6.68	8.54	11.25	13.78	15.69
f_{bin3}	82.52	87.44	87.84	90.23	89.68	89.10	88.49

$\sigma_{bias} = 0.3$

% difference from ground truth

	MC	DM	ID-MC1	ID-MC3	ID-MC5	ID-MC7	ID-MC10
$E[D_{iso}]$	17.09	30.25	12.35	15.75	15.17	14.87	14.77
$E[D_\Delta^2]$	30.77	13.95	20.41	22.14	23.55	24.51	25.51
$E[R_1]$	5.73	7.23	4.62	5.88	5.72	5.55	5.38
$E[R_2]$	3.48	8.20	1.81	0.09	1.33	2.08	2.78
$V[D_{iso}]$	40.81	33.90	14.47	23.09	27.63	30.64	33.72
$V[D_\Delta^2]$	54.35	47.49	46.40	46.81	47.15	47.36	47.61
$V[R_1]$	47.49	118.61	59.57	51.14	46.81	44.38	42.40
$V[R_2]$	16.23	38.04	21.72	8.45	0.79	6.45	12.09
$\Delta_{\omega/2\pi}E[D_{iso}]$	104.00	105.45	72.46	103.10	102.37	101.81	101.25
$\Delta_{\omega/2\pi}E[D_\Delta^2]$	56.61	50.60	27.63	57.81	58.77	59.29	59.92
f_{bin1}	35.99	11.37	21.62	22.80	23.73	24.31	24.84
f_{bin2}	30.08	15.43	15.73	14.40	14.55	14.96	15.49
f_{bin3}	82.52	14.69	44.78	58.03	64.96	69.07	72.97

$\sigma_{bias} = 0.4$

% difference from ground truth

	MC	DM	ID-MC1	ID-MC3	ID-MC5	ID-MC7	ID-MC10
$E[D_{iso}]$	12.35	34.05	12.27	10.84	10.39	10.53	11.05
$E[D_\Delta^2]$	30.77	36.19	5.62	30.88	29.03	28.01	27.39
$E[R_1]$	4.62	39.22	14.37	10.52	8.55	7.23	6.06
$E[R_2]$	3.48	10.41	1.61	0.17	1.09	1.78	2.48
$V[D_{iso}]$	40.81	51.01	22.65	48.12	46.26	43.63	40.38
$V[D_\Delta^2]$	54.35	62.15	56.33	56.19	56.67	56.85	56.74
$V[R_1]$	47.49	20.18	1.96	37.30	42.88	45.78	47.36
$V[R_2]$	16.23	34.23	30.53	14.04	4.25	2.75	9.49
$\Delta_{\omega/2\pi}E[D_{iso}]$	72.46	57.55	36.30	70.04	70.72	71.15	71.72
$\Delta_{\omega/2\pi}E[D_\Delta^2]$	27.63	24.60	21.88	22.46	25.95	28.56	31.47
f_{bin1}	35.99	39.47	0.36	33.23	32.58	32.16	31.63
f_{bin2}	30.08	47.07	40.09	27.09	26.72	26.94	27.46
f_{bin3}	82.52	94.27	64.78	90.77	88.58	85.29	80.33

$\sigma_{bias} = 0.5$

% difference from ground truth

	MC	DM	ID-MC1	ID-MC3	ID-MC5	ID-MC7	ID-MC10
$E[D_{iso}]$	12.35	9.44	7.68	6.01	4.65	3.80	3.32
$E[D_\Delta^2]$	52.11	59.47	30.77	51.88	49.89	48.50	47.15
$E[R_1]$	4.62	10.26	0.36	10.87	8.41	6.33	4.18
$E[R_2]$	3.48	14.07	9.17	3.39	1.44	0.31	2.13
$V[D_{iso}]$	40.81	25.04	37.51	16.83	19.71	21.20	21.89
$V[D_\Delta^2]$	94.27	95.11	54.35	97.26	97.53	97.74	98.09
$V[R_1]$	47.49	7.32	26.44	7.51	5.15	16.15	27.83
$V[R_2]$	16.23	61.13	74.35	47.71	30.01	13.76	3.58
$\Delta_{\omega/2\pi}E[D_{iso}]$	84.14	87.63	72.46	87.41	87.04	87.01	87.17
$\Delta_{\omega/2\pi}E[D_\Delta^2]$	78.30	82.33	27.63	82.29	80.92	79.75	78.82
f_{bin1}	99.96	100.00	35.99	100.00	100.00	100.00	100.00
f_{bin2}	114.50	126.43	30.08	127.20	127.51	127.77	128.34
f_{bin3}	82.52	91.10	61.87	66.70	71.89	74.60	75.87

Supplementary Figure S3: Percent difference from ground truth of mean E[x] and variance V[x] parameters for the different methods under different dictionary bias conditions at SNR of 90.

$\sigma_{bias} = 0.2$

% difference from ground truth

	MC	DM	ID-MC1	ID-MC3	ID-MC5	ID-MC7	ID-MC10
$E[D_{iso}]$	15.07	10.15	9.50	9.52	10.31	11.13	11.84
$E[D_{\Delta}^2]$	36.74	19.33	9.80	21.30	23.25	24.93	26.29
$E[R_1]$	4.39	9.97	0.20	2.54	3.18	3.39	3.46
$E[R_2]$	5.01	1.84	0.27	0.69	1.98	2.98	3.65
$V[D_{iso}]$	42.83	34.04	23.84	41.07	43.58	44.68	45.28
$V[D_{\Delta}^2]$	55.36	57.76	52.83	57.87	57.93	57.99	57.98
$V[R_1]$	56.89	103.09	73.13	59.07	53.85	51.49	50.29
$V[R_2]$	21.59	3.52	8.56	0.69	8.23	14.90	19.22
$\Delta_{\omega/2\pi}E[D_{iso}]$	94.04	93.86	93.58	93.92	94.05	94.18	94.39
$\Delta_{\omega/2\pi}E[D_{\Delta}^2]$	46.13	46.24	25.03	48.03	49.15	49.67	50.11
f_{bin1}	35.76	23.65	12.46	25.38	27.06	28.52	29.59
f_{bin2}	30.86	12.37	5.07	12.86	15.36	17.67	19.49
f_{bin3}	85.96	85.96	82.53	88.36	88.77	88.51	88.44

$\sigma_{bias} = 0.3$

% difference from ground truth

	MC	DM	ID-MC1	ID-MC3	ID-MC5	ID-MC7	ID-MC10
$E[D_{iso}]$	18.50	29.67	15.07	17.48	17.05	16.83	16.78
$E[D_{\Delta}^2]$	36.74	24.51	14.69	26.68	28.44	29.70	31.22
$E[R_1]$	5.48	7.35	4.39	5.69	5.50	5.31	5.06
$E[R_2]$	5.01	8.01	1.31	0.78	2.20	3.11	3.95
$V[D_{iso}]$	42.83	26.70	17.53	26.63	31.58	34.63	37.81
$V[D_{\Delta}^2]$	55.36	47.67	47.20	47.86	48.47	48.98	49.51
$V[R_1]$	56.89	118.48	64.19	57.40	53.95	52.02	50.70
$V[R_2]$	21.59	35.93	19.55	5.85	4.11	10.88	16.96
$\Delta_{\omega/2\pi}E[D_{iso}]$	104.52	104.66	94.04	104.27	104.09	104.05	104.06
$\Delta_{\omega/2\pi}E[D_{\Delta}^2]$	56.10	52.06	25.03	56.76	57.21	57.51	57.77
f_{bin1}	35.76	24.64	11.68	26.23	27.42	28.25	29.25
f_{bin2}	30.86	21.24	15.03	20.39	20.87	21.47	22.27
f_{bin3}	82.53	51.70	20.88	63.77	69.98	73.66	77.36

$\sigma_{bias} = 0.4$

% difference from ground truth

	MC	DM	ID-MC1	ID-MC3	ID-MC5	ID-MC7	ID-MC10
$E[D_{iso}]$	15.07	34.18	14.29	13.51	13.27	13.39	13.84
$E[D_{\Delta}^2]$	36.74	39.06	5.54	34.53	33.30	32.69	32.52
$E[R_1]$	15.13	39.43	4.39	11.24	9.36	8.02	6.68
$E[R_2]$	5.01	10.29	1.28	0.69	1.91	2.78	3.65
$V[D_{iso}]$	42.83	47.79	21.67	44.27	44.18	43.10	41.60
$V[D_{\Delta}^2]$	56.78	62.24	55.36	57.42	57.84	58.06	58.10
$V[R_1]$	56.89	17.73	3.34	38.15	45.40	49.77	53.56
$V[R_2]$	21.59	33.21	28.89	11.91	0.77	6.98	14.67
$\Delta_{\omega/2\pi}E[D_{iso}]$	94.04	59.40	37.04	72.04	73.53	74.69	76.15
$\Delta_{\omega/2\pi}E[D_{\Delta}^2]$	25.03	25.79	22.74	21.73	24.70	27.01	29.51
f_{bin1}	35.76	41.87	0.67	35.97	35.41	35.17	35.06
f_{bin2}	48.03	49.55	30.86	35.48	34.24	33.92	34.13
f_{bin3}	82.53	88.15	67.01	83.00	83.04	81.64	79.66

$\sigma_{bias} = 0.5$

% difference from ground truth

	MC	DM	ID-MC1	ID-MC3	ID-MC5	ID-MC7	ID-MC10
$E[D_{iso}]$	15.07	9.42	7.71	7.66	6.58	5.90	5.54
$E[D_{\Delta}^2]$	51.58	59.48	36.74	51.79	49.87	48.54	47.18
$E[R_1]$	4.39	10.17	0.50	11.25	8.94	6.96	4.81
$E[R_2]$	5.01	13.99	9.36	3.19	1.10	0.75	2.68
$V[D_{iso}]$	42.83	24.13	36.43	16.16	18.95	20.31	21.20
$V[D_{\Delta}^2]$	93.94	95.00	55.36	96.99	97.30	97.53	97.84
$V[R_1]$	56.89	7.10	25.82	7.85	4.91	15.67	27.64
$V[R_2]$	21.59	59.79	73.55	46.46	28.60	12.10	5.87
$\Delta_{\omega/2\pi}E[D_{iso}]$	94.04	88.08	85.76	88.33	88.05	88.28	88.64
$\Delta_{\omega/2\pi}E[D_{\Delta}^2]$	78.33	82.34	25.03	82.09	80.71	79.56	78.59
f_{bin1}	99.10	99.99	35.76	99.99	99.99	99.98	99.97
f_{bin2}	112.12	126.05	30.86	124.25	124.90	125.01	125.17
f_{bin3}	82.53	88.65	62.03	67.39	70.97	72.90	73.81

Supplementary Figure S4: Percent difference from ground truth of mean E[x] and variance V[x] parameters for the different methods under different dictionary bias conditions at SNR of 60.

$\sigma_{bias} = 0.2$

% difference from ground truth

	MC	DM	ID-MC1	ID-MC3	ID-MC5	ID-MC7	ID-MC10
$E[D_{iso}]$	24.03	12.87	10.64	15.90	18.09	19.57	20.65
$E[D_{\Delta}^2]$	42.68	26.91	11.82	29.37	31.80	34.01	35.98
$E[R_1]$	4.09	9.23	0.20	3.40	4.09	4.17	4.04
$E[R_2]$	8.79	1.87	0.04	2.07	4.22	5.79	6.84
$V[D_{iso}]$	34.23	31.59	27.19	35.67	38.23	39.79	40.92
$V[D_{\Delta}^2]$	56.45	54.47	54.23	57.21	57.81	58.23	58.56
$V[R_1]$	66.98	105.78	74.95	63.52	59.23	57.68	57.14
$V[R_2]$	40.05	4.99	2.72	10.07	23.03	31.87	37.62
$\Delta_{\omega/2\pi}E[D_{iso}]$	130.30	104.99	101.47	105.76	106.52	107.30	108.07
$\Delta_{\omega/2\pi}E[D_{\Delta}^2]$	40.62	46.60	21.00	41.95	42.72	43.45	43.73
f_{bin1}	30.84	26.77	13.90	28.01	29.61	31.12	32.48
f_{bin2}	32.63	20.80	2.57	23.90	26.89	29.30	31.35
f_{bin3}	82.77	88.73	70.04	82.67	82.62	82.69	82.85

$\sigma_{bias} = 0.3$

% difference from ground truth

	MC	DM	ID-MC1	ID-MC3	ID-MC5	ID-MC7	ID-MC10
$E[D_{iso}]$	24.03	28.21	23.84	24.43	24.78	24.97	25.18
$E[D_{\Delta}^2]$	42.68	32.52	16.70	35.03	37.33	39.20	41.54
$E[R_1]$	5.82	7.58	4.09	6.29	6.14	5.82	5.37
$E[R_2]$	8.79	7.33	0.77	3.65	5.53	6.73	7.82
$V[D_{iso}]$	34.23	19.03	7.44	26.53	31.37	34.51	37.64
$V[D_{\Delta}^2]$	54.23	50.00	48.83	51.15	52.10	52.93	53.88
$V[R_1]$	66.98	114.97	67.00	62.15	59.55	58.52	58.01
$V[R_2]$	40.05	25.79	8.03	8.49	20.42	28.34	35.34
$\Delta_{\omega/2\pi}E[D_{iso}]$	130.30	104.91	103.67	105.33	105.79	106.32	107.07
$\Delta_{\omega/2\pi}E[D_{\Delta}^2]$	55.07	54.29	21.00	55.35	55.42	55.43	55.32
f_{bin1}	30.84	29.12	12.37	30.74	32.26	33.64	35.30
f_{bin2}	32.63	34.53	15.23	35.01	35.78	36.72	37.91
f_{bin3}	70.04	57.54	39.32	65.58	70.57	73.58	76.59

$\sigma_{bias} = 0.4$

% difference from ground truth

	MC	DM	ID-MC1	ID-MC3	ID-MC5	ID-MC7	ID-MC10
$E[D_{iso}]$	24.03	34.94	20.34	21.81	22.63	23.27	24.01
$E[D_{\Delta}^2]$	42.68	44.07	4.65	41.23	40.97	41.11	41.79
$E[R_1]$	17.96	40.26	4.09	14.03	12.21	10.83	9.22
$E[R_2]$	8.79	9.76	0.34	2.51	4.56	6.01	7.41
$V[D_{iso}]$	34.23	31.79	14.55	27.76	29.82	31.21	32.70
$V[D_{\Delta}^2]$	58.14	62.84	54.23	59.81	59.99	60.15	60.27
$V[R_1]$	66.98	11.27	7.29	34.08	42.74	48.67	54.61
$V[R_2]$	40.05	28.43	21.35	1.50	12.59	22.85	32.96
$\Delta_{\omega/2\pi}E[D_{iso}]$	130.30	63.29	36.76	76.19	78.00	79.58	81.63
$\Delta_{\omega/2\pi}E[D_{\Delta}^2]$	21.00	22.72	20.95	18.64	20.98	22.94	25.26
f_{bin1}	30.84	44.89	0.38	40.13	39.52	39.42	39.56
f_{bin2}	72.44	55.40	32.63	61.40	58.35	56.71	55.17
f_{bin3}	70.04	63.62	67.87	61.62	63.71	65.12	66.68

$\sigma_{bias} = 0.5$

% difference from ground truth

	MC	DM	ID-MC1	ID-MC3	ID-MC5	ID-MC7	ID-MC10
$E[D_{iso}]$	24.03	8.94	7.82	15.04	15.32	15.38	15.54
$E[D_{\Delta}^2]$	45.50	58.69	42.68	49.15	47.17	45.63	43.76
$E[R_1]$	4.09	9.77	1.32	13.01	11.13	9.45	7.49
$E[R_2]$	8.79	14.06	10.23	1.97	0.69	2.82	5.00
$V[D_{iso}]$	34.23	25.70	35.95	12.93	15.52	17.19	18.22
$V[D_{\Delta}^2]$	87.87	93.35	54.23	94.69	95.05	95.29	95.50
$V[R_1]$	66.98	5.09	23.21	7.67	3.91	13.54	24.77
$V[R_2]$	40.05	53.61	68.73	37.27	18.61	2.55	14.66
$\Delta_{\omega/2\pi}E[D_{iso}]$	130.30	91.94	90.88	92.21	92.41	92.71	93.15
$\Delta_{\omega/2\pi}E[D_{\Delta}^2]$	78.37	82.89	21.00	81.36	80.33	79.31	78.26
f_{bin1}	81.00	97.70	30.84	95.22	94.07	93.04	91.59
f_{bin2}	98.46	123.45	32.63	103.30	103.79	103.70	102.68
f_{bin3}	70.04	72.20	86.67	65.65	67.13	67.80	67.23

Colour scale: 20, 40, 60, 80, 100, 120

Supplementary Figure S5: Percent difference from ground truth of mean E[x] and variance V[x] parameters for the different methods under different dictionary bias conditions at SNR of 30.

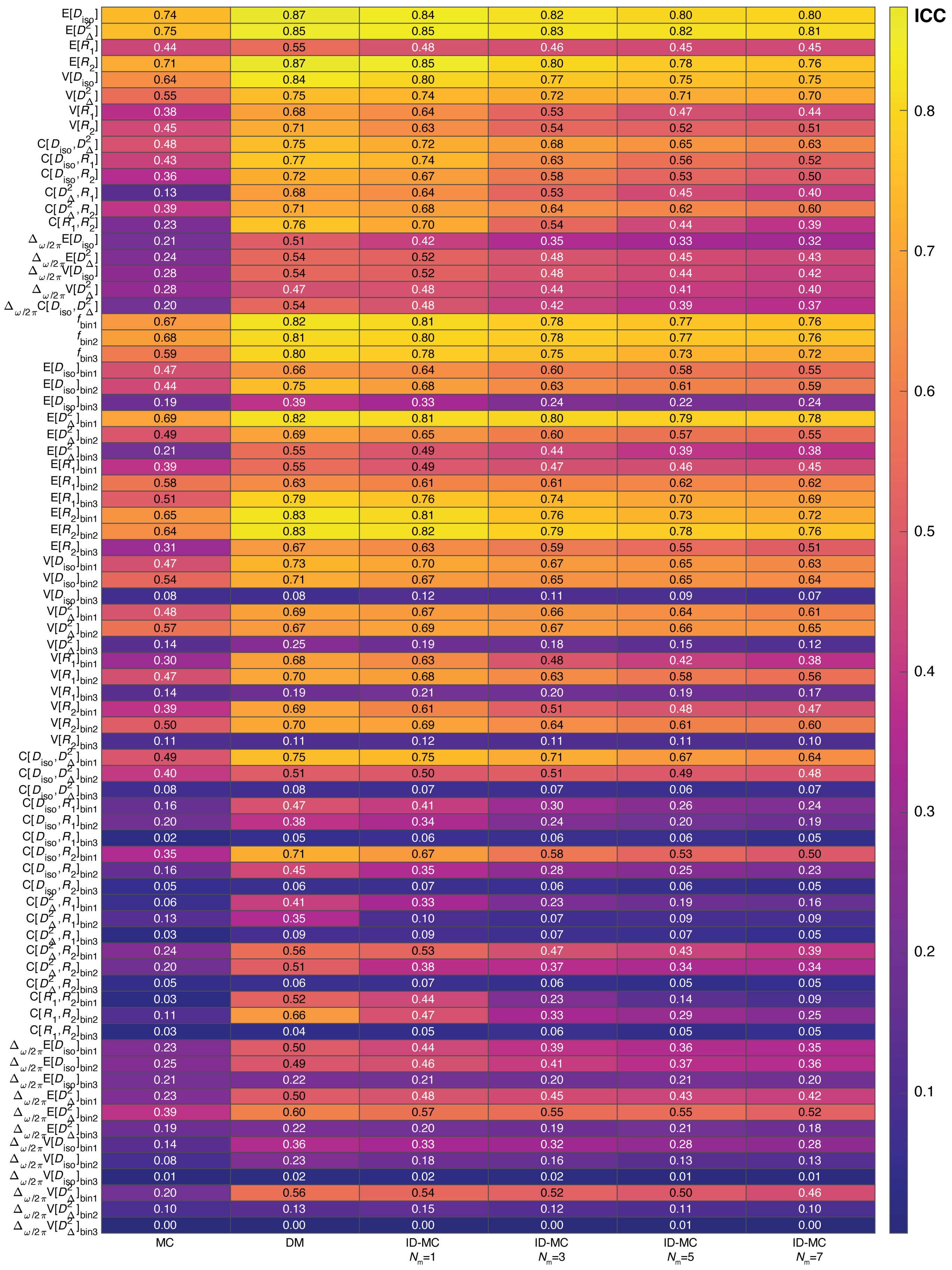

Supplementary Figure S6: Intraclass Correlation Coefficient (ICC) heatmap of median voxel-wise parameter estimates across different inversion methods. Each row corresponds to an MD-MRI parameter, while each column represents an inversion strategy: MC, DM, and ID-MC with different mutation levels (N_m = 1 to N_m = 7). The color scale reflects ICC values, with yellow indicating higher reproducibility and purple indicating lower reproducibility. Negative ICC values were zeroed.

	MC	DM	ID-MC N_m=1	ID-MC N_m=3	ID-MC N_m=5	ID-MC N_m=7
$E[D_{iso}]$	0.01	0.01	0.01	0.01	0.01	0.01
$E[D_{\Delta}^2]$	0.02	0.01	0.01	0.01	0.01	0.01
$E[R_1]$	0.02	0.02	0.02	0.02	0.02	0.02
$E[R_2]$	0.02	0.01	0.01	0.02	0.02	0.02
$V[D_{iso}]$	0.06	0.04	0.04	0.04	0.04	0.04
$V[D_{\Delta}^2]$	0.05	0.02	0.02	0.02	0.03	0.03
$V[R_1]$	0.14	0.05	0.06	0.09	0.10	0.10
$V[R_2]$	0.09	0.04	0.05	0.06	0.07	0.07
$C[D_{iso},D_{\Delta}^2]$	0.29	0.15	0.16	0.18	0.20	0.21
$C[D_{iso},R_1]$	0.24	0.13	0.13	0.16	0.20	0.23
$C[D_{iso},R_2]$	0.21	0.17	0.21	0.19	0.20	0.21
$C[D_{\Delta}^2,R_1]$	1.12	0.14	0.18	0.22	0.29	0.35
$C[D_{\Delta}^2,R_2]$	0.39	0.10	0.13	0.17	0.20	0.22
$C[R_1,R_2]$	0.59	0.12	0.15	0.25	0.29	0.34
$\Delta_{\omega/2\pi}E[D_{iso}]$	1.03	0.11	0.13	0.16	0.18	0.20
$\Delta_{\omega/2\pi}E[D_{\Delta}^2]$	0.08	0.03	0.04	0.04	0.04	0.05
$\Delta_{\omega/2\pi}V[D_{iso}]$	0.85	0.32	0.43	0.50	0.49	0.48
$\Delta_{\omega/2\pi}V[D_{\Delta}^2]$	0.43	0.15	0.13	0.14	0.16	0.18
$\Delta_{\omega/2\pi}C[D_{iso},D_{\Delta}^2]$	1.75	0.21	0.27	0.37	0.51	0.61
f_{bin1}	0.02	0.01	0.01	0.01	0.01	0.01
f_{bin2}	0.05	0.03	0.03	0.04	0.05	0.04
f_{bin3}	0.11	0.17	0.15	0.12	0.11	0.10
$E[D_{iso}]_{bin1}$	0.02	0.01	0.01	0.02	0.02	0.02
$E[D_{iso}]_{bin2}$	0.03	0.02	0.02	0.02	0.02	0.02
$E[D_{iso}]_{bin3}$	0.04	0.01	0.01	0.01	0.01	0.02
$E[D_{\Delta}^2]_{bin1}$	0.01	0.01	0.01	0.01	0.01	0.01
$E[D_{\Delta}^2]_{bin2}$	0.02	0.02	0.01	0.01	0.02	0.02
$E[D_{\Delta}^2]_{bin3}$	0.03	0.01	0.02	0.02	0.02	0.02
$E[R_1]_{bin1}$	0.03	0.02	0.02	0.03	0.03	0.03
$E[R_1]_{bin2}$	0.05	0.02	0.03	0.03	0.03	0.03
$E[R_1]_{bin3}$	0.17	0.15	0.16	0.15	0.15	0.14
$E[R_2]_{bin1}$	0.02	0.01	0.01	0.02	0.02	0.02
$E[R_2]_{bin2}$	0.04	0.02	0.02	0.02	0.03	0.03
$E[R_2]_{bin3}$	0.21	0.22	0.22	0.19	0.19	0.17
$V[D_{iso}]_{bin1}$	0.06	0.02	0.03	0.04	0.04	0.05
$V[D_{iso}]_{bin2}$	0.12	0.07	0.09	0.09	0.08	0.08
$V[D_{iso}]_{bin3}$	0.24	0.66	0.33	0.25	0.31	0.29
$V[D_{\Delta}^2]_{bin1}$	0.05	0.03	0.03	0.03	0.04	0.03
$V[D_{\Delta}^2]_{bin2}$	0.08	0.07	0.06	0.06	0.07	0.06
$V[D_{\Delta}^2]_{bin3}$	1.01	1.67	0.87	0.49	0.94	1.63
$V[R_1]_{bin1}$	0.17	0.05	0.06	0.09	0.10	0.11
$V[R_1]_{bin2}$	0.17	0.10	0.11	0.13	0.15	0.16
$V[R_1]_{bin3}$	1.48	2.10	0.99	0.97	1.00	1.05
$V[R_2]_{bin1}$	0.11	0.04	0.06	0.09	0.09	0.10
$V[R_2]_{bin2}$	0.17	0.10	0.13	0.13	0.14	0.14
$V[R_2]_{bin3}$	1.68	2.24	1.01	1.14	0.79	1.22
$C[D_{iso},D_{\Delta}^2]_{bin1}$	0.14	0.05	0.06	0.08	0.08	0.09
$C[D_{iso},D_{\Delta}^2]_{bin2}$	0.20	0.11	0.16	0.15	0.17	0.16
$C[D_{iso},D_{\Delta}^2]_{bin3}$	3.76	5.07	1.23	0.49	1.99	1.65
$C[D_{iso},R_1]_{bin1}$	0.61	0.15	0.22	0.51	0.58	0.68
$C[D_{iso},R_1]_{bin2}$	0.47	1.06	0.52	0.62	0.71	0.68
$C[D_{iso},R_1]_{bin3}$	4.42	4.47	3.14	3.16	4.38	3.63
$C[D_{iso},R_2]_{bin1}$	0.23	0.08	0.09	0.12	0.14	0.16
$C[D_{iso},R_2]_{bin2}$	1.44	0.45	0.64	1.00	1.32	1.45
$C[D_{iso},R_2]_{bin3}$	4.47	4.47				
$C[D_{\Delta}^2,R_1]_{bin1}$	0.69	0.15	0.26	0.64	0.97	1.25
$C[D_{\Delta}^2,R_1]_{bin2}$	0.64	0.31	2.47	2.59	3.46	2.74
$C[D_{\Delta}^2,R_1]_{bin3}$	4.47	4.47			3.45	
$C[D_{\Delta}^2,R_2]_{bin1}$	0.28	0.11	0.13	0.16	0.18	0.20
$C[D_{\Delta}^2,R_2]_{bin2}$	0.41	0.20	0.30	0.27	0.33	0.32
$C[D_{\Delta}^2,R_2]_{bin3}$	4.47	4.47	4.47	4.47		
$C[R_1,R_2]_{bin1}$	1.34	0.33	0.61	0.88	1.30	1.24
$C[R_1,R_2]_{bin2}$	0.63	0.16	0.29	0.37	0.40	0.40
$C[R_1,R_2]_{bin3}$	3.70	4.45	4.47	3.68	4.47	4.41
$\Delta_{\omega/2\pi}E[D_{iso}]_{bin1}$	0.54	0.13	0.15	0.17	0.20	0.21
$\Delta_{\omega/2\pi}E[D_{iso}]_{bin2}$	0.97	0.15	0.15	0.18	0.20	0.22
$\Delta_{\omega/2\pi}E[D_{iso}]_{bin3}$	0.33	0.19	0.17	0.18	0.19	0.16
$\Delta_{\omega/2\pi}E[D_{\Delta}^2]_{bin1}$	0.10	0.04	0.05	0.05	0.06	0.06
$\Delta_{\omega/2\pi}E[D_{\Delta}^2]_{bin2}$	0.15	0.07	0.06	0.06	0.06	0.07
$\Delta_{\omega/2\pi}E[D_{\Delta}^2]_{bin3}$	0.14	0.07	0.08	0.08	0.11	0.09
$\Delta_{\omega/2\pi}V[D_{iso}]_{bin1}$	0.43	0.18	0.19	0.26	0.28	0.29
$\Delta_{\omega/2\pi}V[D_{iso}]_{bin2}$	0.85	0.36	0.50	0.48	0.58	0.41
$\Delta_{\omega/2\pi}V[D_{iso}]_{bin3}$	2.72	3.63	3.29	2.16	2.25	2.20
$\Delta_{\omega/2\pi}V[D_{\Delta}^2]_{bin1}$	0.29	0.09	0.11	0.14	0.14	0.16
$\Delta_{\omega/2\pi}V[D_{\Delta}^2]_{bin2}$	0.41	0.56	0.49	0.55	0.78	1.22
$\Delta_{\omega/2\pi}V[D_{\Delta}^2]_{bin3}$	1.21	3.33	2.01	1.60	1.79	1.65

CV_{ws} colour scale: 10^{-2}, 10^{-1}, 10^{0}; black = NaN

Supplementary Figure S7: Within-subject coefficient of variation (CV_{ws}) heatmap of ROI-based parameter estimates across different inversion methods. Each row corresponds to an MD-MRI parameter, while each column represents an inversion strategy: MC, DM, and ID-MC with different mutation levels (N_m = 1 to N_m = 7). The color scale reflects CV_{ws} values, with purple indicating higher reproducibility and yellow indicating lower reproducibility.

Parameter	MC	DM	ID-MC N_m=1	ID-MC N_m=3	ID-MC N_m=5	ID-MC N_m=7
$\mathrm{E}[D_{\mathrm{iso}}]$	0.03	0.02	0.02	0.02	0.03	0.03
$\mathrm{E}[D_\Delta^2]$	0.04	0.03	0.03	0.03	0.03	0.03
$\mathrm{E}[R_1]$	0.05	0.03	0.04	0.04	0.04	0.05
$\mathrm{E}[R_2]$	0.04	0.02	0.02	0.03	0.03	0.03
$\mathrm{V}[D_{\mathrm{iso}}]$	0.15	0.09	0.10	0.11	0.11	0.11
$\mathrm{V}[D_\Delta^2]$	0.11	0.05	0.06	0.06	0.07	0.07
$\mathrm{V}[R_1]$	0.28	0.10	0.11	0.16	0.19	0.22
$\mathrm{V}[R_2]$	0.20	0.10	0.13	0.16	0.17	0.18
$\mathrm{C}[D_{\mathrm{iso}},D_\Delta^2]$	0.57	0.37	0.38	0.40	0.42	0.43
$\mathrm{C}[D_{\mathrm{iso}},R_1]$	0.60	0.31	0.31	0.40	0.47	0.51
$\mathrm{C}[D_{\mathrm{iso}},R_2]$	0.45	0.39	0.47	0.48	0.50	0.50
$\mathrm{C}[D_\Delta^2,R_1]$	2.29	0.45	0.72	0.80	0.95	1.06
$\mathrm{C}[D_\Delta^2,R_2]$	0.75	0.22	0.28	0.34	0.37	0.40
$\mathrm{C}[R_1,R_2]$	1.50	0.31	0.52	0.71	0.83	0.91
$\Delta_{\omega/2\pi}\mathrm{E}[D_{\mathrm{iso}}]$	1.32	0.17	0.21	0.27	0.30	0.33
$\Delta_{\omega/2\pi}\mathrm{E}[D_\Delta^2]$	0.18	0.08	0.08	0.09	0.10	0.11
$\Delta_{\omega/2\pi}\mathrm{V}[D_{\mathrm{iso}}]$	1.20	0.44	0.48	0.55	0.61	0.67
$\Delta_{\omega/2\pi}\mathrm{V}[D_\Delta^2]$	0.97	0.55	0.52	0.56	0.59	0.60
$\Delta_{\omega/2\pi}\mathrm{C}[D_{\mathrm{iso}},D_\Delta^2]$	1.58	0.34	0.40	0.51	0.60	0.67
f_{bin1}	0.06	0.03	0.03	0.04	0.04	0.04
f_{bin2}	0.15	0.07	0.08	0.09	0.10	0.10
f_{bin3}	0.84	0.64	0.66	0.67	0.70	0.71
$\mathrm{E}[D_{\mathrm{iso}}]_{\mathrm{bin1}}$	0.04	0.02	0.02	0.02	0.03	0.03
$\mathrm{E}[D_{\mathrm{iso}}]_{\mathrm{bin2}}$	0.07	0.04	0.04	0.05	0.05	0.05
$\mathrm{E}[D_{\mathrm{iso}}]_{\mathrm{bin3}}$	0.07	0.02	0.02	0.03	0.03	0.04
$\mathrm{E}[D_\Delta^2]_{\mathrm{bin1}}$	0.02	0.01	0.01	0.01	0.02	0.02
$\mathrm{E}[D_\Delta^2]_{\mathrm{bin2}}$	0.04	0.03	0.03	0.03	0.03	0.03
$\mathrm{E}[D_\Delta^2]_{\mathrm{bin3}}$	0.07	0.03	0.04	0.05	0.05	0.05
$\mathrm{E}[R_1]_{\mathrm{bin1}}$	0.06	0.03	0.04	0.05	0.05	0.05
$\mathrm{E}[R_1]_{\mathrm{bin2}}$	0.12	0.05	0.06	0.07	0.08	0.08
$\mathrm{E}[R_1]_{\mathrm{bin3}}$	0.93	0.65	0.69	0.71	0.76	0.77
$\mathrm{E}[R_2]_{\mathrm{bin1}}$	0.04	0.02	0.03	0.03	0.03	0.03
$\mathrm{E}[R_2]_{\mathrm{bin2}}$	0.12	0.04	0.04	0.06	0.06	0.07
$\mathrm{E}[R_2]_{\mathrm{bin3}}$	1.17	0.88	0.93	0.97	1.03	1.07
$\mathrm{V}[D_{\mathrm{iso}}]_{\mathrm{bin1}}$	0.14	0.07	0.08	0.09	0.10	0.10
$\mathrm{V}[D_{\mathrm{iso}}]_{\mathrm{bin2}}$	0.29	0.16	0.20	0.21	0.21	0.21
$\mathrm{V}[D_{\mathrm{iso}}]_{\mathrm{bin3}}$	1.06	1.68	1.06	1.03	1.04	1.06
$\mathrm{V}[D_\Delta^2]_{\mathrm{bin1}}$	0.14	0.07	0.07	0.08	0.08	0.09
$\mathrm{V}[D_\Delta^2]_{\mathrm{bin2}}$	0.33	0.17	0.21	0.22	0.22	0.22
$\mathrm{V}[D_\Delta^2]_{\mathrm{bin3}}$	1.37	1.72	1.29	1.26	1.29	1.32
$\mathrm{V}[R_1]_{\mathrm{bin1}}$	0.34	0.11	0.12	0.16	0.20	0.23
$\mathrm{V}[R_1]_{\mathrm{bin2}}$	0.47	0.18	0.24	0.28	0.33	0.35
$\mathrm{V}[R_1]_{\mathrm{bin3}}$	2.64	2.52	2.41	2.46	2.49	2.55
$\mathrm{V}[R_2]_{\mathrm{bin1}}$	0.25	0.12	0.15	0.19	0.21	0.21
$\mathrm{V}[R_2]_{\mathrm{bin2}}$	0.46	0.21	0.27	0.31	0.34	0.35
$\mathrm{V}[R_2]_{\mathrm{bin3}}$	2.76	2.95	2.82	2.78	2.83	2.87
$\mathrm{C}[D_{\mathrm{iso}},D_\Delta^2]_{\mathrm{bin1}}$	0.25	0.12	0.13	0.14	0.16	0.17
$\mathrm{C}[D_{\mathrm{iso}},D_\Delta^2]_{\mathrm{bin2}}$	0.41	0.22	0.28	0.29	0.29	0.29
$\mathrm{C}[D_{\mathrm{iso}},D_\Delta^2]_{\mathrm{bin3}}$	2.26	3.13	2.48	2.20	2.26	2.32
$\mathrm{C}[D_{\mathrm{iso}},R_1]_{\mathrm{bin1}}$	1.16	0.41	0.48	0.74	0.95	1.08
$\mathrm{C}[D_{\mathrm{iso}},R_1]_{\mathrm{bin2}}$	1.57	1.51	1.41	1.57	1.71	1.78
$\mathrm{C}[D_{\mathrm{iso}},R_1]_{\mathrm{bin3}}$	4.43	4.18	4.15	4.19	4.19	4.29
$\mathrm{C}[D_{\mathrm{iso}},R_2]_{\mathrm{bin1}}$	0.35	0.21	0.23	0.26	0.27	0.28
$\mathrm{C}[D_{\mathrm{iso}},R_2]_{\mathrm{bin2}}$	2.05	0.80	1.43	1.63	1.78	1.82
$\mathrm{C}[D_{\mathrm{iso}},R_2]_{\mathrm{bin3}}$	3.97	3.96	3.94	3.98	4.04	4.03
$\mathrm{C}[D_\Delta^2,R_1]_{\mathrm{bin1}}$	1.26	0.47	0.65	1.11	1.39	1.52
$\mathrm{C}[D_\Delta^2,R_1]_{\mathrm{bin2}}$	1.77	0.77	3.35	3.80	3.73	3.69
$\mathrm{C}[D_\Delta^2,R_1]_{\mathrm{bin3}}$	4.34	3.47	3.53	3.75	3.85	3.95
$\mathrm{C}[D_\Delta^2,R_2]_{\mathrm{bin1}}$	0.51	0.28	0.28	0.32	0.35	0.39
$\mathrm{C}[D_\Delta^2,R_2]_{\mathrm{bin2}}$	1.10	0.42	0.68	0.76	0.81	0.83
$\mathrm{C}[D_\Delta^2,R_2]_{\mathrm{bin3}}$	3.79	3.57	3.48	3.61	3.78	3.84
$\mathrm{C}[R_1,R_2]_{\mathrm{bin1}}$	2.41	1.04	1.45	1.93	2.10	2.20
$\mathrm{C}[R_1,R_2]_{\mathrm{bin2}}$	1.81	0.41	0.85	1.06	1.12	1.21
$\mathrm{C}[R_1,R_2]_{\mathrm{bin3}}$	3.58	3.55	3.52	3.47	3.53	3.53
$\Delta_{\omega/2\pi}\mathrm{E}[D_{\mathrm{iso}}]_{\mathrm{bin1}}$	0.71	0.20	0.22	0.27	0.30	0.32
$\Delta_{\omega/2\pi}\mathrm{E}[D_{\mathrm{iso}}]_{\mathrm{bin2}}$	1.43	0.35	0.33	0.38	0.43	0.46
$\Delta_{\omega/2\pi}\mathrm{E}[D_{\mathrm{iso}}]_{\mathrm{bin3}}$	0.67	0.73	0.69	0.66	0.62	0.63
$\Delta_{\omega/2\pi}\mathrm{E}[D_\Delta^2]_{\mathrm{bin1}}$	0.22	0.09	0.10	0.11	0.12	0.13
$\Delta_{\omega/2\pi}\mathrm{E}[D_\Delta^2]_{\mathrm{bin2}}$	0.32	0.20	0.18	0.18	0.19	0.20
$\Delta_{\omega/2\pi}\mathrm{E}[D_\Delta^2]_{\mathrm{bin3}}$	0.45	0.34	0.37	0.38	0.37	0.37
$\Delta_{\omega/2\pi}\mathrm{V}[D_{\mathrm{iso}}]_{\mathrm{bin1}}$	1.02	0.53	0.54	0.58	0.60	0.61
$\Delta_{\omega/2\pi}\mathrm{V}[D_{\mathrm{iso}}]_{\mathrm{bin2}}$	2.02	1.19	1.30	1.40	1.47	1.52
$\Delta_{\omega/2\pi}\mathrm{V}[D_{\mathrm{iso}}]_{\mathrm{bin3}}$	3.96	3.88	3.97	3.98	3.93	3.99
$\Delta_{\omega/2\pi}\mathrm{V}[D_\Delta^2]_{\mathrm{bin1}}$	0.55	0.24	0.25	0.28	0.30	0.33
$\Delta_{\omega/2\pi}\mathrm{V}[D_\Delta^2]_{\mathrm{bin2}}$	1.24	1.82	1.74	2.04	2.36	2.57
$\Delta_{\omega/2\pi}\mathrm{V}[D_\Delta^2]_{\mathrm{bin3}}$	2.87	3.78	3.37	3.28	3.14	3.13

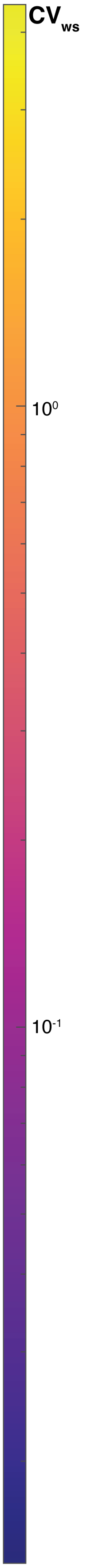

Supplementary Figure S8: Within-subject coefficient of variation (CV_{ws}) heatmap of median voxel-wise parameter estimates across different inversion methods. Each row corresponds to an MD-MRI parameter, while each column represents an inversion strategy: MC, DM, and ID-MC with different mutation levels (N_m = 1 to N_m = 7). The color scale reflects CV_{ws} values, with purple indicating higher reproducibility and yellow indicating lower reproducibility.

A. $E[D_{iso}]$ (m^2/s)

	MC	DM	ID-MC1	ID-MC3	ID-MC5	ID-MC7
Basal Ganglia	8.8e-10	8.4e-10	8.5e-10	8.5e-10	8.5e-10	8.4e-10
Thalamus	9.8e-10	9.1e-10	9.4e-10	9.3e-10	9.4e-10	9.1e-10
MOG	9.2e-10	8.9e-10	9.0e-10	8.9e-10	8.9e-10	8.9e-10
IFG	1.1e-09	1.0e-09	1.0e-09	1.0e-09	1.0e-09	1.0e-09
CC genu	1.1e-09	1.1e-09	1.1e-09	1.1e-09	1.1e-09	1.1e-09
CC body	1.1e-09	1.1e-09	1.1e-09	1.1e-09	1.1e-09	1.1e-09
CC Splenium	1.1e-09	1.1e-09	1.1e-09	1.1e-09	1.1e-09	1.1e-09
Internal Capsule	9.9e-10	9.6e-10	9.4e-10	9.5e-10	9.6e-10	9.6e-10
Corona Radiata	1.1e-09	1.0e-09	1.0e-09	1.0e-09	1.0e-09	1.0e-09
Sagittal Stratum	1.2e-09	1.2e-09	1.1e-09	1.2e-09	1.2e-09	1.2e-09
External Capsule	9.9e-10	9.5e-10	9.6e-10	9.5e-10	9.5e-10	9.4e-10

B. $E[D_\Delta^2]$

	MC	DM	ID-MC1	ID-MC3	ID-MC5	ID-MC7
Basal Ganglia	3.3e-01	2.3e-01	2.4e-01	2.6e-01	2.6e-01	2.7e-01
Thalamus	3.8e-01	3.2e-01	3.3e-01	3.4e-01	3.4e-01	3.5e-01
MOG	3.2e-01	2.1e-01	2.2e-01	2.4e-01	2.5e-01	2.5e-01
IFG	2.5e-01	1.6e-01	1.7e-01	1.8e-01	1.8e-01	1.9e-01
CC genu	5.5e-01	5.2e-01	5.5e-01	5.4e-01	5.4e-01	5.3e-01
CC body	5.7e-01	5.5e-01	5.6e-01	5.6e-01	5.7e-01	5.7e-01
CC Splenium	6.4e-01	6.2e-01	6.3e-01	6.4e-01	6.4e-01	6.5e-01
Internal Capsule	6.4e-01	5.8e-01	6.0e-01	6.1e-01	6.2e-01	6.2e-01
Corona Radiata	5.6e-01	5.1e-01	5.2e-01	5.3e-01	5.4e-01	5.4e-01
Sagittal Stratum	4.7e-01	4.7e-01	4.7e-01	4.7e-01	4.7e-01	4.7e-01
External Capsule	4.5e-01	4.0e-01	4.1e-01	4.2e-01	4.3e-01	4.3e-01

C. $E[R_1]$ (1/s)

	MC	DM	ID-MC1	ID-MC3	ID-MC5	ID-MC7
Basal Ganglia	9.6e-01	9.8e-01	9.6e-01	9.6e-01	9.6e-01	9.6e-01
Thalamus	9.8e-01	1.0e+00	9.9e-01	9.8e-01	9.9e-01	9.9e-01
MOG	7.9e-01	8.1e-01	7.9e-01	7.9e-01	7.9e-01	7.9e-01
IFG	7.1e-01	7.4e-01	7.1e-01	7.1e-01	7.1e-01	7.1e-01
CC genu	1.1e+00	1.1e+00	1.1e+00	1.1e+00	1.1e+00	1.1e+00
CC body	1.0e+00	1.1e+00	1.0e+00	1.0e+00	1.0e+00	1.0e+00
CC Splenium	1.1e+00	1.2e+00	1.1e+00	1.1e+00	1.1e+00	1.2e+00
Internal Capsule	1.1e+00	1.1e+00	1.1e+00	1.1e+00	1.1e+00	1.1e+00
Corona Radiata	1.1e+00	1.1e+00	1.1e+00	1.1e+00	1.1e+00	1.1e+00
Sagittal Stratum	1.2e+00	1.2e+00	1.2e+00	1.2e+00	1.2e+00	1.2e+00
External Capsule	1.0e+00	1.0e+00	1.0e+00	9.9e-01	1.0e+00	1.0e+00

D. $E[R_2]$ (1/s)

	MC	DM	ID-MC1	ID-MC3	ID-MC5	ID-MC7
Basal Ganglia	2.1e+01	2.1e+01	2.1e+01	2.1e+01	2.1e+01	2.1e+01
Thalamus	1.8e+01	1.8e+01	1.8e+01	1.8e+01	1.8e+01	1.8e+01
MOG	1.7e+01	1.7e+01	1.7e+01	1.7e+01	1.7e+01	1.7e+01
IFG	1.5e+01	1.5e+01	1.5e+01	1.5e+01	1.5e+01	1.5e+01
CC genu	1.7e+01	1.7e+01	1.7e+01	1.7e+01	1.7e+01	1.7e+01
CC body	1.5e+01	1.5e+01	1.5e+01	1.5e+01	1.5e+01	1.5e+01
CC Splenium	1.6e+01	1.6e+01	1.6e+01	1.6e+01	1.6e+01	1.6e+01
Internal Capsule	1.8e+01	1.8e+01	1.8e+01	1.8e+01	1.8e+01	1.7e+01
Corona Radiata	1.5e+01	1.6e+01	1.5e+01	1.5e+01	1.5e+01	1.5e+01
Sagittal Stratum	1.6e+01	1.6e+01	1.6e+01	1.6e+01	1.6e+01	1.6e+01
External Capsule	1.7e+01	1.7e+01	1.7e+01	1.7e+01	1.7e+01	1.7e+01

E. $\Delta_{\omega/2\pi}E[D_{iso}]$ (m^2)

	MC	DM	ID-MC1	ID-MC3	ID-MC5	ID-MC7
Basal Ganglia	6.7e-13	4.0e-12	2.8e-12	3.3e-12	3.0e-12	3.7e-12
Thalamus	-5.5e-13	3.8e-12	3.4e-12	2.8e-12	2.5e-12	2.3e-12
MOG	5.6e-12	8.0e-12	8.2e-12	8.0e-12	7.8e-12	7.6e-12
IFG	-2.7e-13	4.3e-12	3.1e-12	3.5e-12	3.2e-12	3.9e-12
CC genu	-2.5e-12	1.5e-12	6.3e-13	9.6e-13	7.6e-13	1.3e-12
CC body	-9.1e-13	2.6e-12	1.7e-12	2.0e-12	1.8e-12	2.3e-12
CC Splenium	1.2e-12	2.4e-12	2.3e-12	2.0e-12	1.9e-12	1.8e-12
Internal Capsule	7.2e-13	3.2e-12	2.1e-12	2.5e-12	2.3e-12	3.0e-12
Corona Radiata	1.3e-13	4.2e-12	4.0e-12	3.5e-12	3.2e-12	3.0e-12
Sagittal Stratum	-1.5e-12	1.5e-12	1.3e-12	9.6e-13	7.9e-13	6.7e-13
External Capsule	-8.7e-13	2.6e-12	1.5e-12	1.9e-12	1.6e-12	2.3e-12

Supplementary Figure S9: Mean of region-of-interest-averaged MD-MRI parameters, obtained using the different processing approaches. Abbreviations: MOG, middle occipital gyrus; IFG, inferior frontal gyrus; CC genu, genu of the corpus callosum; CC body, body of the corpus callosum; CC splenium, splenium of the corpus callosum.

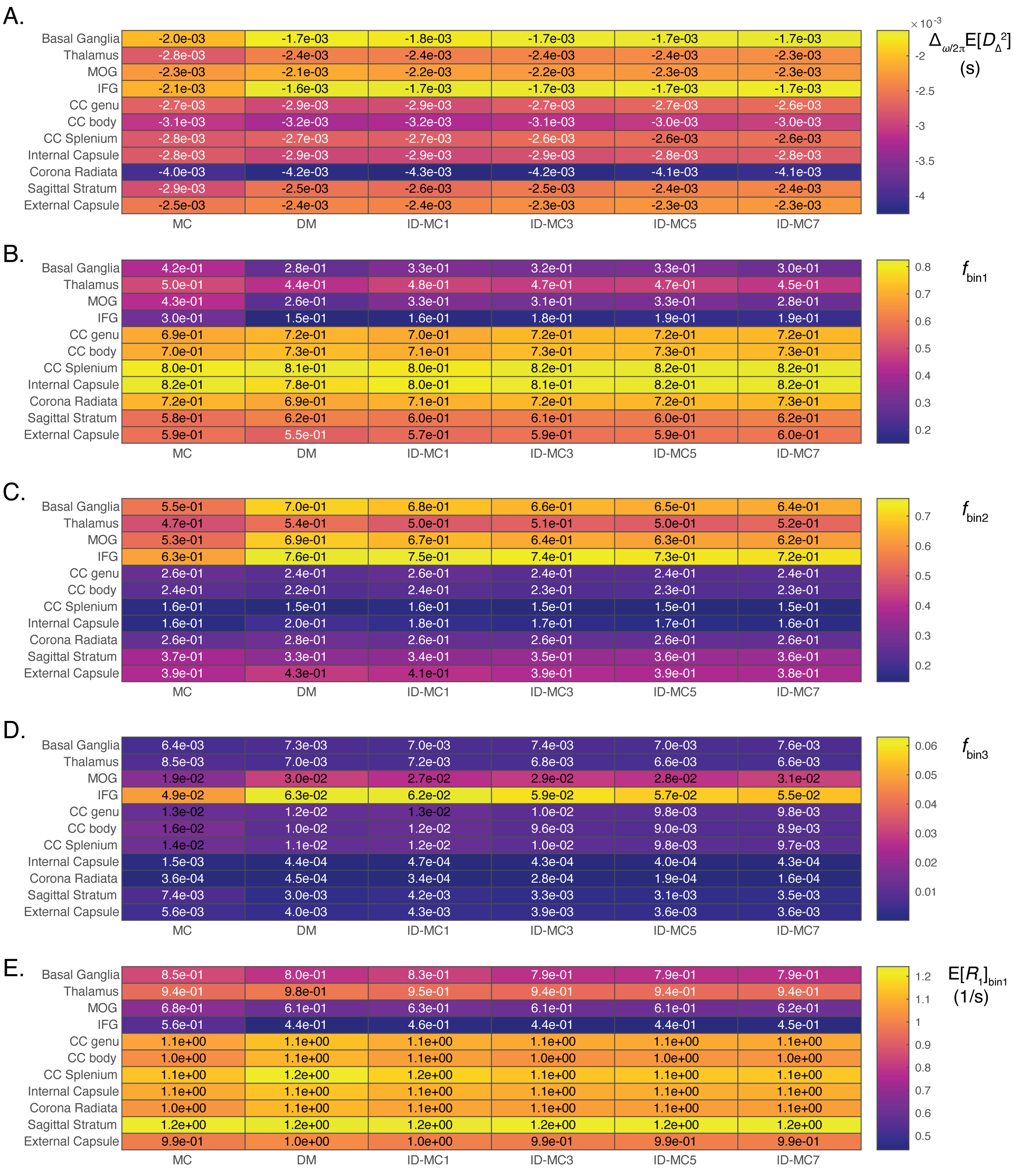

Supplementary Figure S10: Mean of region-of-interest-averaged MD-MRI parameters, obtained using the different processing approaches. Abbreviations: MOG, middle occipital gyrus; IFG, inferior frontal gyrus; CC genu, genu of the corpus callosum; CC body, body of the corpus callosum; CC splenium, splenium of the corpus callosum.

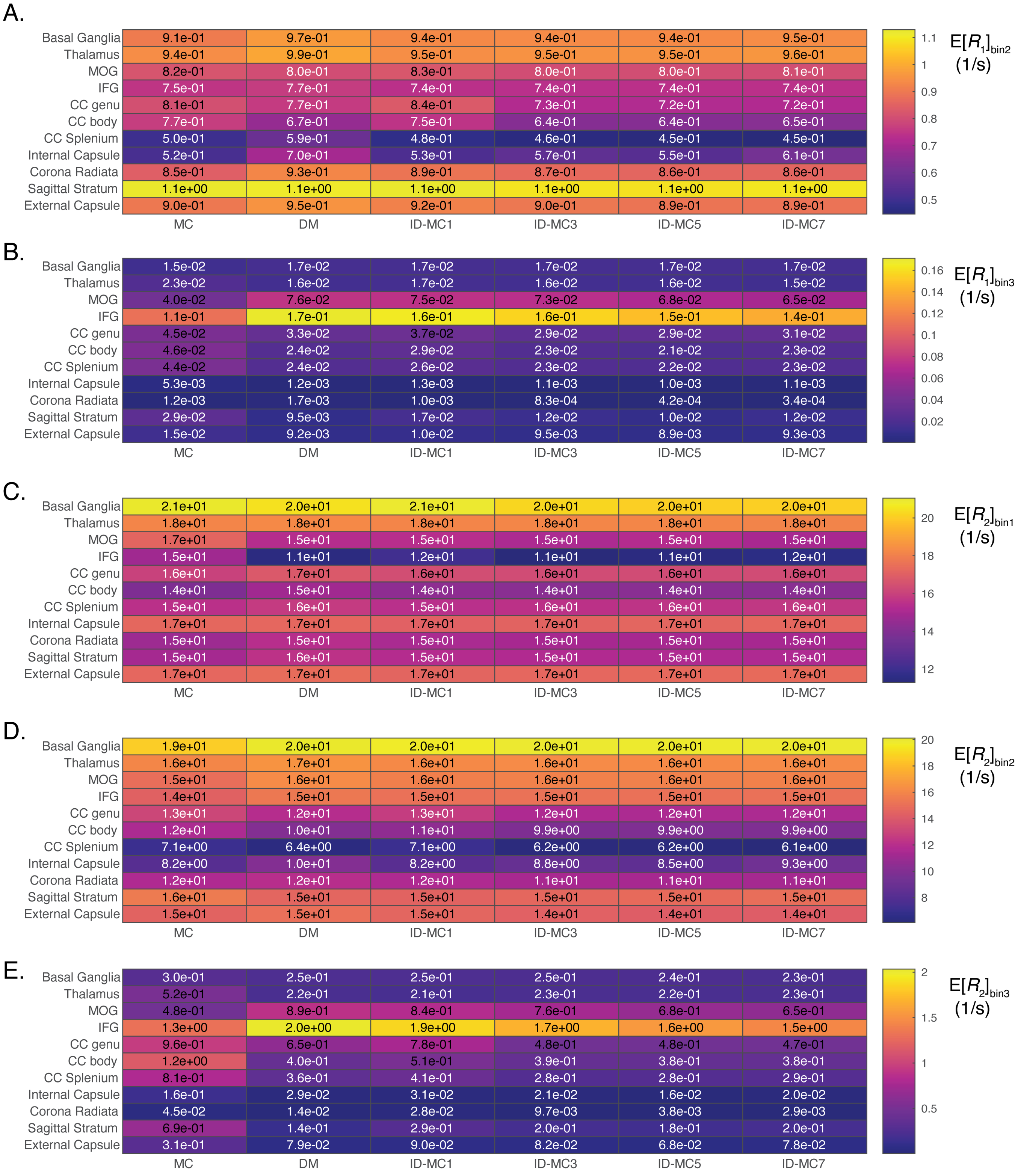

Supplementary Figure S11: Mean of region-of-interest-averaged MD-MRI parameters, obtained using the different processing approaches. Abbreviations: MOG, middle occipital gyrus; IFG, inferior frontal gyrus; CC genu, genu of the corpus callosum; CC body, body of the corpus callosum; CC splenium, splenium of the corpus callosum.

Supplementary Tables

Supplementary Table S1: Comprehensive summary of the in vivo MD-MRI acquisition protocol.

Parameter	Value / Description
Scanner	3T MAGNETOM Prisma (Siemens Healthcare, Erlangen, Germany)
Head coil	32-channel receive-only
Structural scan	Fat-suppressed T_1-weighted MPRAGE (1 mm isotropic)
Sequence	Single-shot spin-echo EPI with tensor-valued diffusion encoding
Voxel size	2 mm isotropic
Field of view (FOV)	$228 \times 228 \times 110$ mm^3
Bandwidth	1512 Hz/Px
Parallel imaging	GRAPPA (R = 2), 24 reference lines
Echo spacing	0.8 ms
Partial Fourier	0.75 (phase-encoding direction)
Phase encoding	AP (plus one reversed PA $b = 0$ for distortion correction)
Repetition times (TR)	0.62, 1.75, 3.5, 5, 7, 7.6 s
Echo times (TE)	40, 63, 83, 150 ms
Diffusion weighting (b)	0.1–3 ms/μm^2
B-tensor shapes	Linear ($b_\Delta = 1$), Planar ($b_\Delta = -0.5$), Spherical ($b_\Delta = 0$)
Frequency range	Centroid $\omega_{\text{cent}}/2\pi = 6.6$–$21$ Hz
Number of volumes	139 unique diffusion-relaxation measurements
Scan time	40 minutes
Reference	Johnson et al.[1]

Supplementary Table S2: Dictionary simulation components for bias-controlled experiments.

Component \ Parameter	$D_{\parallel}$ [ms/μm^2]	$D_{\perp}$ [ms/μm^2]	θ [°]	ϕ [°]	D_0 [ms/μm^2]	$\Gamma_{\parallel}$ [rad/s]	$\Gamma_{\perp}$ [rad/s]	R_1 [1/s]	R_2 [1/s]	w
					$\lambda_{\mathbf{bias}} = 0.2$					
**I**	0.5	0.4	0	0	2.1	219	293	1.0	18	0.30
**II**	2.0	0.1	0.9	0.8	2.4	170	688	1.5	15	0.25
**III**	2.8	0.1	0.7	2.5	2	130	825	1.6	12	0.27
**IV**	2.6	2.9	0	0	4	11800	10367	0.3	4	0.16
					$\lambda_{\mathbf{bias}} = 0.3$					
**I**	0.6	0.7	0	0	2.0	149	293	0.9	9	0.29
**II**	2.8	0.06	0.8	0.6	1.6	138	888	2.0	14	0.30
**III**	3.0	0.03	0.8	2.5	1.2	279	636	1.8	18	0.21
**IV**	4.6	3.8	0	0	3.2	8740	13088	0.2	5	0.19
					$\lambda_{\mathbf{bias}} = 0.4$					
**I**	0.4	0.7	0	0	3.1	126	281	0.3	11	0.25
**II**	3.4	0.1	0.6	1.2	2.0	24	815	1.0	10	0.32
**III**	1.9	0.1	2.0	0.8	3.4	173	538	1.0	19	0.25
**IV**	3.2	5.4	0	0	3.2	12854	10576	4.3	4	0.17
					$\lambda_{\mathbf{bias}} = 0.5$					
**I**	0.7	0.7	0	0	2.2	280	505	1.2	12	0.47
**II**	2.7	0.1	1.5	0.8	1.2	280	1023	0.5	25	0.1
**III**	3.1	0.1	0.6	4	2	221	793	1.2	10	0.30
**IV**	4.0	3	0	0	0.9	14398	13208	0.4	6	0.15

References

[1] Johnson, J.T.E., Irfanoglu, M.O., Manninen, E., Ross, T.J., Yang, Y., Laun, F.B., Martin, J., Topgaard, D., Benjamini, D., 2024. In vivo disentanglement of diffusion frequency-dependence, tensor shape, and relaxation using multidimensional MRI. Human Brain Mapping 45, e26697. doi:10.1002/hbm.26697.
